# Improvement in phosphate acquisition and utilization by a secretory purple acid phosphatase (OsPAP21b) in rice

**DOI:** 10.1111/pbi.12699

**Published:** 2017-03-02

**Authors:** Poonam Mehra, Bipin Kumar Pandey, Jitender Giri

**Affiliations:** ^1^ National Institute of Plant Genome Research New Delhi India

**Keywords:** acid phosphatase, organic phosphates, P use efficiency, overexpression, RNAi, root, secretory protein

## Abstract

Phosphate (Pi) deficiency in soil system is a limiting factor for rice growth and yield. Majority of the soil phosphorus (P) is organic in nature, not readily available for root uptake. Low Pi‐inducible purple acid phosphatases (PAPs) are hypothesized to enhance the availability of Pi in soil and cellular system. However, information on molecular and physiological roles of rice PAPs is very limited. Here, we demonstrate the role of a novel rice PAP, OsPAP21b in improving plant utilization of organic‐P. *OsPAP21b* was found to be under the transcriptional control of OsPHR2 and strictly regulated by plant Pi status at both transcript and protein levels. Biochemically, OsPAP21b showed hydrolysis of several organophosphates at acidic pH and possessed sufficient thermostability befitting for high‐temperature rice ecosystems with acidic soils. Interestingly, OsPAP21b was revealed to be a secretory PAP and encodes a distinguishable major APase (acid phosphatase) isoform under low Pi in roots. Further, *OsPAP21b*‐overexpressing transgenics showed increased biomass, APase activity and P content in both hydroponics supplemented with organic‐P sources and soil containing organic manure as sole P source. Additionally, overexpression lines depicted increased root length, biomass and lateral roots under low Pi while RNAi lines showed reduced root length and biomass as compared to WT. In the light of these evidences, present study strongly proposes *OsPAP21b* as a useful candidate for improving Pi acquisition and utilization in rice.

## Introduction

Given its key role in metabolism and signalling, phosphorus (P) is essential for plant growth and development. However, plant‐available P (Pi) is often a limiting factor for crop production in many world soils. About 20 mha of upland area under rice cultivation is Pi deficient (Neue *et al*., 1990). In major rice‐producing areas such as India, ~60% soils have low to medium Pi availability (Murumkar *et al*., 2015). Application of phosphatic fertilizers can ameliorate soil Pi deficiency. Unfortunately, the source of Pi fertilizers, rock phosphate is finite, rapidly depleting and concentrated only in few regions worldwide (Cordell *et al*., 2009). Further, applied Pi is quickly fixed into insoluble inorganic or organic forms due to its high reactivity and microbial action (Pandey *et al*., 2013; Richardson and Simpson, 2011). As rice is one of the major consumers of Pi fertilizers, enhancement of P use efficiency is highly desired for sustainable rice production.

Owing to its high reactivity, there exist more than 170 mineral or inorganic forms of P in soil (Holford, 1997). However, only ionic forms (orthophosphates; H_2_PO_4_^−^ and HPO_4_^2−^) of P in soil solution constitute the ‘plant‐available’ P. Further, a large fraction of soil P (50%–80%) occurs as organic‐P and constitutes the bound P pool, not readily available for root uptake (Wang *et al*., 2009). These organic forms majorly include phosphate monoesters (derivatives of inositol hexakisphosphate) and to some extent phosphate diesters (nucleic acids, phospholipids, cyclic phosphates) and phosphonates (George and Richardson, 2008; Turner, 2008). Consequently, despite having significant amount of P in the form of organic complexes in soil, plants experience its deficiency. Plants have devised several strategies to solubilize these P forms which involve exudation of organic acids and protons, modification of root system architecture, association with mycorrhiza and secretion of APases and phytases (Shen *et al*., 2011).

PAPs are involved in Pi acquisition and utilization in plants (Kuang *et al*., 2009). These enzymes represent the largest group of APases (E.C. 3.1.3.2) and are characterized by their pink or purple colour in water solution due to a charge transfer transition between Tyr residue to Fe (III) in binuclear metal centre (Oddie *et al*., 2000; Wang *et al*., 2015). PAPs contain five conserved blocks of seven amino acids (**D**XG/G**D**XX**Y**/**G**NH(D/E)/VXX**H**/G**H**X**H**) which coordinate the metal binding at binuclear centre (Li *et al*., 2002). PAPs are present in diverse organisms including bacteria, fungi, plants and mammals (Kuang *et al*., 2009). Plant PAPs exist as multigene family in Arabidopsis (Li *et al*., 2002), rice (Zhang *et al*., 2011), soybean (Li *et al*., 2012a) and maize (González‐Muñoz *et al*., 2015). These PAPs catalyse the hydrolysis of several P‐containing organic compounds at acidic pH (pH 4–7). Intracellular PAPs remobilize Pi from cellular reserves, whereas secreted ones hydrolyse P compounds in apoplast and rhizosphere. Several PAPs have been reported to be induced by Pi deficiency in Arabidopsis and crop plants (González‐Muñoz *et al*., 2015; Li *et al*., 2002; Mehra *et al*., 2016; Zhang *et al*., 2011). Overexpression of few of them could improve the plant growth on organic‐P‐supplemented media (*reviewed in* Tian and Liao, 2015). In Arabidopsis, 29 putative PAPs have been reported, of which 11 are low Pi inducible (Wang *et al*., 2014). Some of these PAPs, *AtPAP17*,* AtPAP26*,* AtPAP12*,* AtPAP25*,* AtPAP15* and *AtPAP10*, have been well characterized for their ability to hydrolyse organic‐P (Del Vecchio *et al*., 2014; Kuang *et al*., 2009; del Pozo *et al*., 1999; Tran *et al*., 2010b; Wang *et al*., 2011). In rice, 26 PAPs have been identified, of which ten are induced significantly under Pi deficiency (Mehra *et al*., 2016; Zhang *et al*., 2011). Recently, rice PAPs, *OsPAP10a* and *OsPAP10c*, the low Pi‐inducible rice homologues of *AtPAP10*, were shown to increase ATP hydrolysis when overexpressed in rice (Lu *et al*., 2016; Tian *et al*., 2012). However, except for these two PAPs, no detailed study of any of the rice PAPs has been carried out so far using rice as a system. Further, studies encompassing biochemical properties, transcriptional regulation and loss of function of rice PAPs are largely missing.

Here, we investigated role of a novel PAP, OsPAP21b in improving Pi acquisition and utilization in rice through elaborate biochemical, molecular and functional characterization. Our results revealed that OsPAP21b is a secretory protein and plays key roles in organic‐P utilization in rice.

## Results

### *OsPAP21b* is a low Pi‐induced gene and regulated by OsPHR2

**Figure 1:**
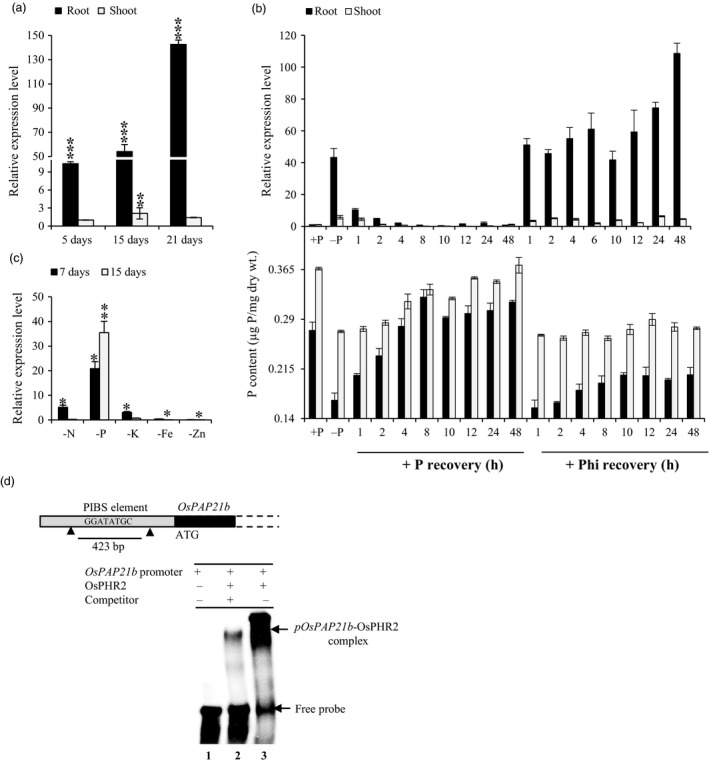
Transcriptional regulation of *OsPAP21b* under Pi deficiency. (a) Expression of *OsPAP21b* in rice under Pi deficiency. Relative expression under Pi deficiency was evaluated with respect to Pi sufficient conditions at 5, 15 and 21 days in WT. (b) Expression of *OsPAP21b* (upper panel) and total P content (lower panel) in roots and shoots of 15‐day‐old seedlings under +P, −P and after recovery of Pi‐starved seedlings with either Pi or Phi. (c) Expression profiling of *OsPAP21b* after 7 and 15 days of nitrogen (−N), phosphorus (−P), potassium (−K), iron (−Fe) and zinc (−Zn) deficiency in roots. Gene expression levels under nutrient‐deficient conditions with respect to corresponding sufficient conditions were determined by qRT‐PCR. *P value <0.05; **P value <0.01; ***P value <0.001 were determined by Student's *t*‐test. (d) Binding of *OsPAP21b* promoter with OsPHR2 by EMSA. 423‐bp promoter region of *OsPAP21b* containing one P1BS element (−421 to −414 bp) was radiolabeled with [α^32^P]CTP and used for binding assays with recombinant OsPHR2 protein. Slow migrating protein–DNA complexes and free probe are indicated by arrow at the top and bottom of the PAGE gel, respectively.

In our previous transcriptome study of two rice genotypes under Pi deficiency, *OsPAP21b* was highly induced especially, in low Pi‐tolerant genotype (Mehra *et al*., 2016). In the present study, we found relatively higher up‐regulation of *OsPAP21b* in roots as compared to shoot tissues under Pi deficiency (Figure 1a). This indicates that *OsPAP21b* is a root‐preferential phosphate starvation response (PSR) gene. We further found that up‐regulation is specific to Pi starvation as prolonged exposure to different nutrient deficiencies led to the induction of *OsPAP21b* in roots under Pi deficiency only (Figure 1c). Although slight up‐regulation of *OsPAP21b* was also observed after 7 days of N and K deficiency, after 15 days, it was down‐regulated. This suggests that *OsPAP21b* is primarily responsive to Pi deficiency. We next analysed the transcriptional regulation of *OsPAP21b* in 15‐day‐old Pi‐starved seedlings resupplied with +Phi (320 μm Phosphite) or +P (320 μm NaH_2_PO_4_). Phi is a non‐metabolizable form of Pi which cannot substitute for Pi in plants, and is known to interfere or suppress the expression of many low Pi‐inducible genes (Varadarajan *et al*., 2002). However, in our study, *OsPAP21b* was found to be largely non‐responsive to Phi treatment as *OsPAP21b* was consistently up‐regulated even after 48 h of Phi supply (Figure 1b). On the other hand, Pi resupply to Pi‐starved seedlings suppressed the expression of *OsPAP21b* within 1 h in roots and 2 h in shoot (Figure 1b). Notably, this suppression was accompanied with simultaneous increase in total P content in these tissues. This implies that induction of *OsPAP21b* strongly depends on Pi status of plants and not by local availability of Pi in media.

#### OsPHR2 physically interacts with the promoter of OsPAP21b

Most of the low Pi‐induced molecular responses in rice are regulated by a MYB transcription factor, OsPHR2. To test whether *OsPAP21b* is also transcriptionally regulated by OsPHR2‐dependent pathway, we scanned 2 kb promoter region of *OsPAP21b* and found one potential OsPHR2 binding site (PIBS element) between −421 and −414 upstream of ATG. The slow migrating protein–DNA complexes in EMSA gel indicated binding of OsPHR2 with *OsPAP21b* promoter (Figure 1d). Further, competitive EMSA with 400‐fold excess of unlabelled *OsPAP21b* promoter (competitor) confirmed specificity of this physical interaction (Figure 1d).

### Phylogeny of OsPAP21b with other plant PAPs of Ib subgroup

**Figure 2:**
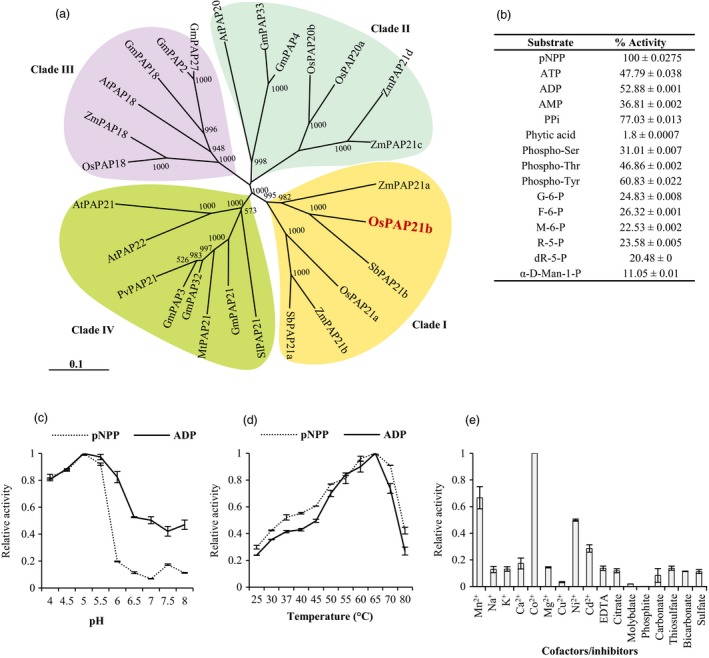
Phylogeny and biochemical properties of OsPAP21b. (a) NJ tree representing phylogenetic relationship of OsPAP21b with other PAPs of Ib subgroup from *Oryza sativa* (OsPAPs), *Arabidopsis thaliana* (AtPAPs), *Zea mays* (ZmPAPs), *Glycine max* (GmPAPs), *Phaseolus vulgaris* (PvPAPs), *Solanum lycopersicum* (SlPAPs), *Medicago truncatula* (MtPAPs) and *Sorghum bicolor* (SbPAPs). Tree was generated in ClustalX 2.0.11 with 1000 bootstrap and viewed with TreeView 1.6. Scale bar represents rate of amino acid substitutions. (b) Relative APase activity of OsPAP21b on different P‐containing substrates. APase activity with pNPP was considered as 100%. (c) Effect of pH and (d) temperature on APase activity of OsPAP21b using pNPP and ADP as substrates. (e) Influence of different anions and divalent cations on APase activity of OsPAP21b using pNPP as substrate. For calculating relative APase activities, maximum activity was considered equal to 1. Values are means from three independent experiments with standard deviations.

PAPs of Ib subgroup were identified in eight different plants including rice and Arabidopsis using NCBI blast search. On the basis of multiple sequence alignments, neighbour‐joining tree was constructed which subdivided all Ib subgroup PAPs into four clades (Figure 2a). Interestingly, OsPAP21b showed closest homology with PAPs of other monocots (*Zea mays* and *Sorghum bicolor*) and formed one clade (Clade I). On the other hand, Arabidopsis homologue of OsPAP21b, AtPAP21 grouped with Ib PAPs of other dicots and formed a distinct clade, clade IV. Other 1b rice PAPs, OsPAP18 and OsPAP20 separated into two different clades (III and II, respectively) which revealed further divergence among rice Ib subgroup PAPs.

### OsPAP21b is a functional acid phosphatase

OsPAP21b had all seven conserved amino acids required for its catalytic activity. To test its activity *in vitro*, we purified recombinant 6xHis‐OsPAP21b protein by immobilized metal‐ion chromatography (IMAC) and analysed on SDS‐PAGE (Figure S1). A band of expected size, i.e. 51.24 kDa, was detected which was further confirmed by immunoblotting with anti‐OsPAP21b antibody (Figure S1d). Enzyme activity assays showed that OsPAP21b can release Pi from different P‐containing organic and inorganic substrates revealing that OsPAP21b is a functional phosphatase with broad substrate specificity (Figure 2b). The highest activity of OsPAP21b was detected with generic substrate pNPP followed by inorganic substrate PPi. Analysis of kinetic parameters revealed that OsPAP21b possesses sufficient specific activity with pNPP (2.0246 ± 0.0932 units/mg protein) and ADP (1.8363 ± 0.2684 units/mg protein) with *K*_*m*_ 0.09 ± 0.01 mm and 0.077 ± 0.004 mm, respectively. Interestingly, OsPAP21b showed fairly high activity with phosphorylated amino acids (p‐Ser, p‐Thr and p‐Tyr) and ADP. Our analysis further confirmed APase nature of OsPAP21b as highest activity was observed in acidic pH (pH 5.0) (Figure 2c). Additionally, OsPAP21b was found to be moderately thermostable with highest activity at 65 °C (Figure 2d). We further investigated the effect of different anions and divalent cations on the activity of OsPAP21b (Figure 2e). Notably, activity of OsPAP21b was completely inhibited by high concentration of Pi and showed 50% activity inhibition (IC_50_) at 5.09 ± 1.34 mm concentration of Pi. Further, Co^2+^, Mn^2+^ and Ni^2+^ were found to be preferred cofactors for OsPAP21b activity.

### Overexpression of *OsPAP21b* improved plant growth on organic‐P substrates

**Figure 3:**
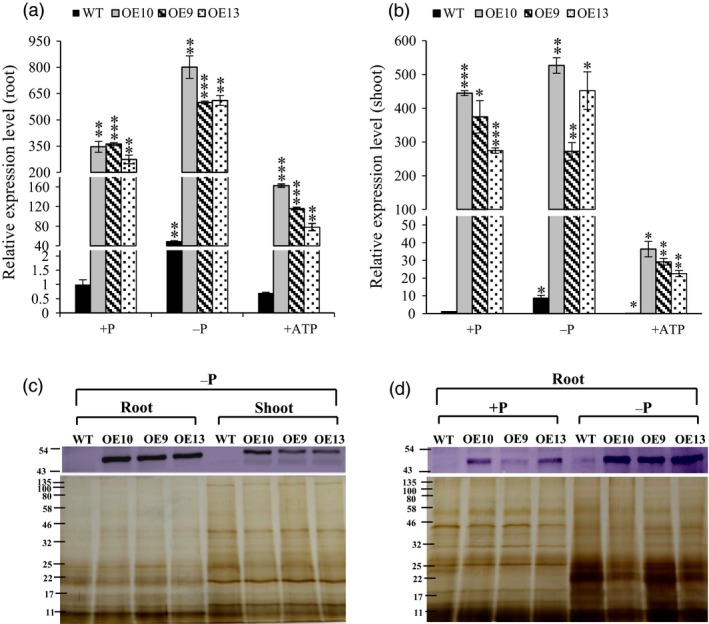
Analysis of *OsPAP21b* transcript and protein levels in OE lines (OE10, 9, 13). (a) Relative expression levels of *OsPAP21b* in roots and (b) shoots of OE lines as compared to WT. All relative expression levels were calculated with respect to WT +P. Plants were raised for 30 days under +P, −P and +ATP (15‐day‐old Pi‐starved seedlings were recovered with ATP for another 15 days) conditions. *P value <0.05; **P value <0.01; ***P value <0.001. (c) Western blot showing increased protein levels of OsPAP21b in 30‐day‐old OE lines relative to WT in roots and shoots under −P condition. (d) Western blot of OsPAP21b in roots of WT and OE lines under +P and −P conditions. 24 μg of total protein was loaded in each lane and resolved on 12% SDS‐PAGE. OsPAP21b accumulation was probed with anti‐OsPAP21b antibody. Silver‐stained protein gels show the equal amount of protein loading in each lane.

To elucidate the functional roles of *OsPAP21b*, full‐length cDNA of *OsPAP21b* along with 5′ and 3′ UTRs was constitutively overexpressed under *ZmUbi1* promoter in rice (OE lines; Figures S2, S3). All OE lines showed significant overexpression of *OsPAP21b* as compared to WT (wild type) at both transcript and protein levels (Figure 3). To assess the effects of *OsPAP21b* overexpression, three independent T3 homozygous lines and WT were grown under +P (320 μm NaH_2_PO_4_), −P (1 μm NaH_2_PO_4_) and +ATP (15‐day‐old −P grown seedlings recovered with 320 μm ATP for the next 15 days) for 30 days. Notably, OE lines and WT showed increased accumulation of both transcripts and protein under −P conditions in roots as compared to their corresponding +P condition (Figure 3a, d). Moreover, relatively higher protein levels were observed in roots as compared to shoots in OE lines under −P condition (Figure 3c). This was consistent with higher up‐regulation of *OsPAP21b* in roots as compared to shoots in OE lines as compared to WT (Figure 3a, b). Further, distinct band of OsPAP21b could not be detected in WT by western blot, indicating low expression of OsPAP21b (Figure 3c, d). However, with higher protein load, a faint band could be visualized in WT under −P condition in roots (Figure 3d).

Interestingly, there was a drastic decrease in the level of transcripts in all ATP supplied OE lines (recovery) as compared to +P (Figure 3a, b). This indicates probable degradation or down‐regulation of *OsPAP21b* transcripts under ATP recovery and points towards post‐transcriptional regulation of *OsPAP21b* in OE lines in Pi status‐dependent manner.

**Figure 4:**
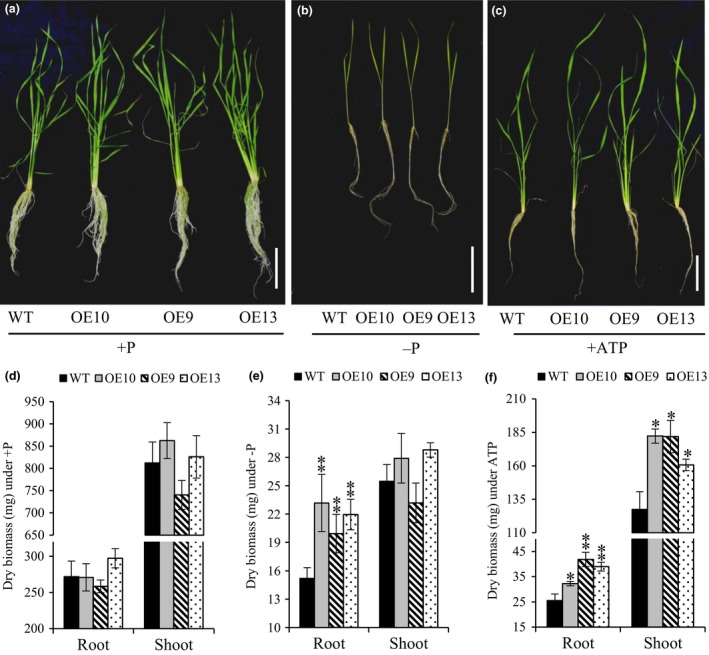
Growth of WT and *OsPAP21b* OE lines (OE10, 9, 13) under different P treatments. (a–c) Phenotype of WT and *OsPAP21b* OE lines under +P, −P and +ATP recovery conditions. scale bar = 10 cm. (d–f) Root and shoot dry biomass. Plants were raised hydroponically for 30 days under +P, −P and +ATP recovery conditions (Pi‐starved seedlings for 15 days were recovered with ATP for 15 subsequent days). Each values represents mean of 15 seedlings in three replicates with standard error. *P value <0.05; **P value <0.01.

**Figure 5:**
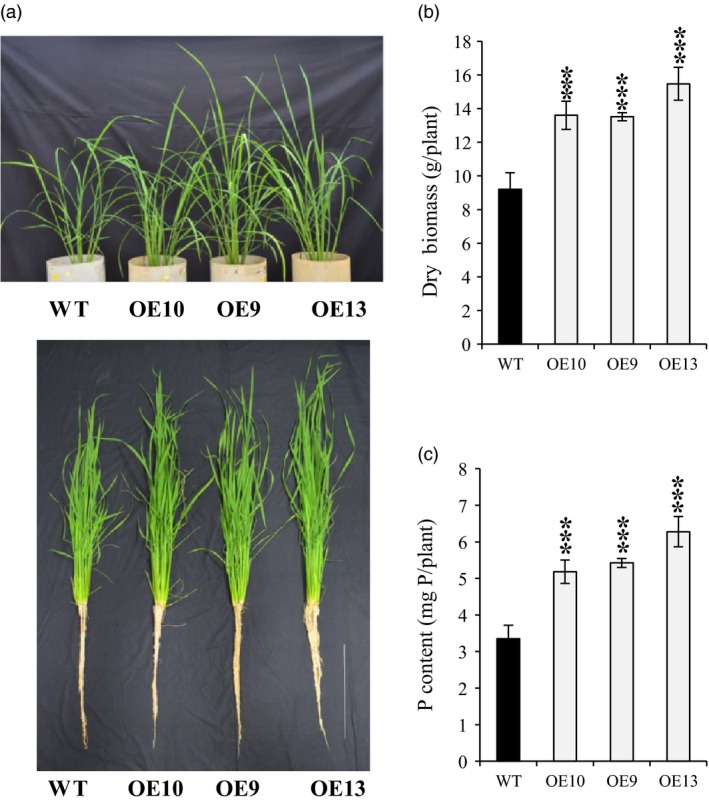
Growth and P content of soil‐grown WT and *OsPAP21b* OE lines. (a) Phenotype of 2‐month‐old WT and *OsPAP21b* OE lines in soils supplemented with manure as organic‐P source. Scale bar =30 cm. (b) Root and shoot dry biomass and (c) total P content per plant of *OsPAP21b* OE lines and WT. Each bar represents average of 3 replicates (*n* = 10) with standard error. ***P value <0.001.

Morphologically, total lateral root length in OE lines was increased by almost 1.6–2 times as compared to WT under −P (Figure S4). Additionally, lateral length/cm of root was significantly increased by 1.2–1.5 times in OE line in relation to WT (Figure S4). These results indicate that *OsPAP21b* influences root system architecture under Pi deficiency by increasing lateral length. Further analysis revealed ~6%–15% increase in root length in OE lines than WT under −P (Figure S5b). However, noticeable advantage (8%–11%) in shoot length was recorded only in ATP recovered Pi‐starved OE lines (Figure S5c). Similarly, OE lines produced higher root and shoot biomass under +ATP as compared to WT (Figure 4c, f). Marked increase in root biomass was also observed in OE lines as compared to WT under −P condition (Figure 4b, e). However, no significant differences in plant biomass were found between WT and OE lines under +P (Figure 4a, d). These results suggest that constitutive overexpression of *OsPAP21b* does not affect the normal plant growth and development under sufficient Pi supply; however, it plays important role in improving growth on organic‐P substrate through better Pi uptake and utilization. We further investigated growth performance of OE lines on organophosphates other than ATP (Figure S6). Similar to ATP recovery, OE lines showed significant increase in root and shoot length and biomass on ADP and p‐Ser as compared to WT (Figure S6). However, on phytate‐supplemented media, significant differences in shoot biomass were observed only in OE9 as compared to WT. Apart from hydroponics, Pi‐starved *OsPAP21b* OE lines also showed better recovery than WT in soil system supplemented with only organic manure as P source (Figure 5a). About 47%–68% increase in plant biomass (Figure 5b) and 54%–87% in P content (Figure 5c) was observed in OE lines as compared to WT. Interestingly, all OE lines also showed early flowering as compared to WT (Figure S7). These results suggest high potential of OsPAP21b in utilizing natural organic‐P sources.

### Overexpression of *OsPAP21b* enhanced APase activity and P content

**Figure 6:**
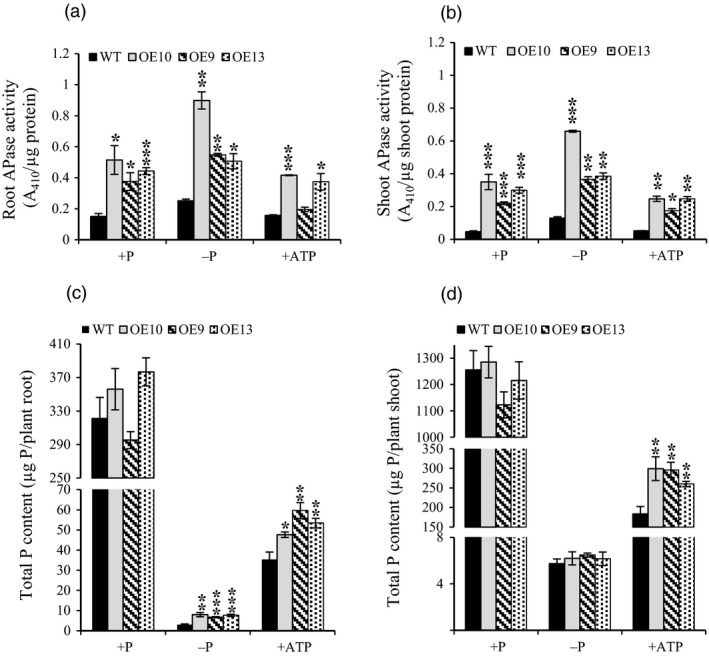
Total APase activity and P content of WT and *OsPAP21b* OE lines. (a) Total APase activity in roots and (b) shoots of 30‐day‐old seedlings raised under +P, −P and +ATP conditions. Activity was determined with 1 μg of total root protein using pNPP as substrate. (c) Total P content in roots and (d) shoots of 30‐day‐old seedlings under +P, −P and +ATP conditions. Each bar represents means of three replicates (*n* = 5) with standard error. *P value <0.05; **P value <0.01; ***P value <0.001.

Total APase activity of 30‐day‐old seedlings was measured under +P, −P and +ATP conditions (Figure 6a, b). Irrespective of treatments, APase activity was fairly high in root as compared to shoot. Under +P, total APase activity was significantly increased by ~2.5‐ to 3.8‐fold in roots and ~5.5‐ to 8.7‐fold in shoots of OE lines as compared to WT. APase activity was further increased in all OE lines and WT in −P condition relative to +P condition. However, in response to ATP recovery, APase activity was significantly decreased in OE lines as compared to both +P and −P conditions. This again reveals Pi‐dependent regulation of OsPAP21b. To assess the effect of increased APase activity on Pi uptake, total P content per plant was quantitated (Figure 6c, d). Significant increase was observed in roots (35%–69%) and shoots (42%–63%) of ATP recovered OE lines as compared to WT. About twofold to 2.5‐fold increase in total P content was also observed in roots of Pi‐starved OE lines in relation to WT. However, no significant increase in total P content was found in OE lines as compared to WT under +P.

### Expression of other PAPs and Pi transporters is altered in *OsPAP21b* transgenics

As OE lines showed higher P accumulation under −P, we studied expression of other low Pi‐inducible PAPs and Pi transporters in roots of WT and OE lines grown under +P, −P and +ATP. Our analysis revealed significant up‐regulation of *OsPAP3b*,* OsPAP10a*,* OsPAP10c*,* OsPAP23* and *OsPAP27a* under Pi deficiency in OE lines as compared to WT (Figure S8). Similarly, significant up‐regulation of Pi transporters *OsPT2*,* OsPT4* and *OsPT9* was also observed in OE lines as compared to WT under Pi deficiency (Figure S9). These results indicate potential signalling roles of *OsPAP21b* and also explain high P accumulation in transgenics.

### *OsPAP21b* encodes a secretory PAP

**Figure 7:**
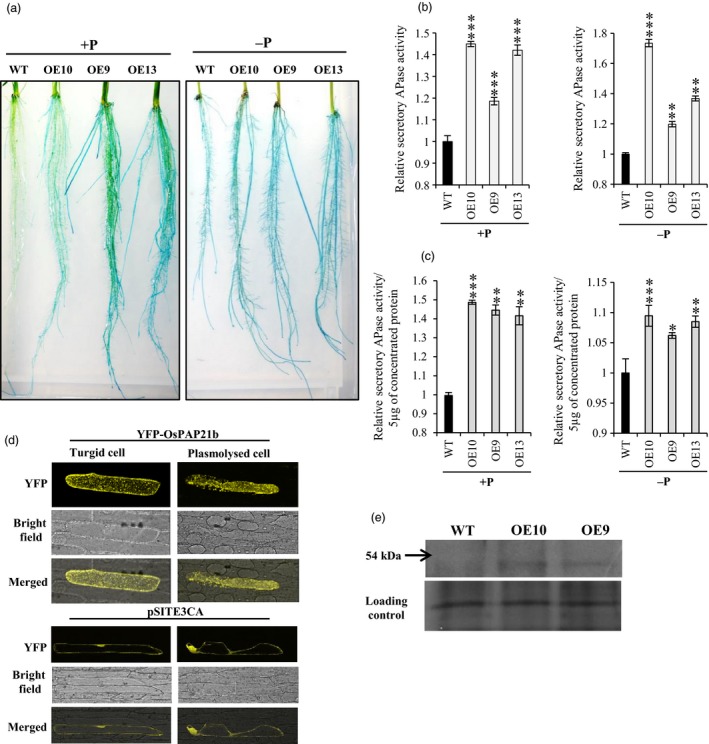
*OsPAP21b* encodes a secretory PAP. (a) Activity staining of roots of 15‐day‐old WT and *OsPAP21b* OE lines under +P and −P. Roots were overlaid with 0.015% BCIP in 0.5% agar and blue colour shows APase activity. (b) Quantitation of secretory APase activity in growth medium of 30‐day‐old seedlings under +P and −P conditions. (c) Quantitation of secretory APase activity in 30‐day‐old seedlings under +P and −P conditions using 5 μg of concentrated total protein secreted into medium. Relative activity was determined with respect to APase activity in WT which was considered equal to one. *P value <0.05; **P value <0.01; ***P value <0.001. (d) YFP fluorescence in turgid and plasmolysed onion epidermal cells overexpressing YFP‐OsPAP21b or only YFP. (e) Detection of OsPAP21b under −P conditions in concentrated media protein of 30‐day‐old WT and OE lines by western blotting. Silver‐stained protein gel shows equal protein load in each lane.

As predicted by SignalP 3.0, OsPAP21b contains a signal peptide at its N terminal end and therefore may be a secretory PAP (Zhang *et al*., 2011). To confirm this, we did multiple experiments. First, secretory APase activity of OsPAP21b was tested by staining of roots using BCIP as substrate. Intense blue colour precipitate was observed on root surfaces of OE lines as compared to WT under +P (Figure 7a). This indicates increased hydrolysis of BCIP into stable coloured indolyl derivative by secreted OsPAP21b in OE lines. However, under −P conditions, apparent differences in activity staining could not be observed between OE lines and WT. This may be due to rapid saturation of colour intensity by diverse APases secreted in response to Pi deficiency. Second, secretory APase activity was measured in plant growth media under +P and −P conditions. Again all OE lines showed 18%–44% increase in APase activity as compared to WT under +P (Figure 7b). Under −P conditions, increase in secretory APase activity was observed as high as 73% in OE10 as compared to WT (Figure 7b). Observed difference in total secretory APase activity could also be attributed to differences in total root surface area. To overrule this, secretory APase activity was also determined with equal amount of concentrated protein from growth media. Notably, all OE lines showed significantly higher (42%–49%) secretory APase activity as compared to WT under +P (Figure 7c). However, under −P, only 6%–10% increase in APase activity could be observed in OE lines as compared to WT (Figure 7c). We further confirmed these results by western blotting of concentrated secreted protein with anti‐OsPAP21b antibody (Figure 7e). Lastly, we confirmed OsPAP21b secretion by plasmolysis of onion epidermal cells overexpressing YFP‐OsPAP21b fusion protein. YFP signals were clearly noticed in apoplast of plasmolysed cells (Figure 7d). Overexpression of YFP‐OsPAP21b in onion cells showed dotted fluorescence pattern all over the cell which indicated its localization in endomembrane system (Figure S10). All this clearly revealed the secretory nature of OsPAP21b. To further determine the localization of OsPAP21b, co‐localization assays were performed with various organelle markers tagged with mCherry (Figure S10). Although perfect overlap between fluorescence signals obtained from OsPAP21b and markers could not be obtained, the pattern of YFP‐OsPAP21b seems to be similar to Golgi marker.

### *OsPAP21b* encodes a major APase isoform

**Figure 8:**
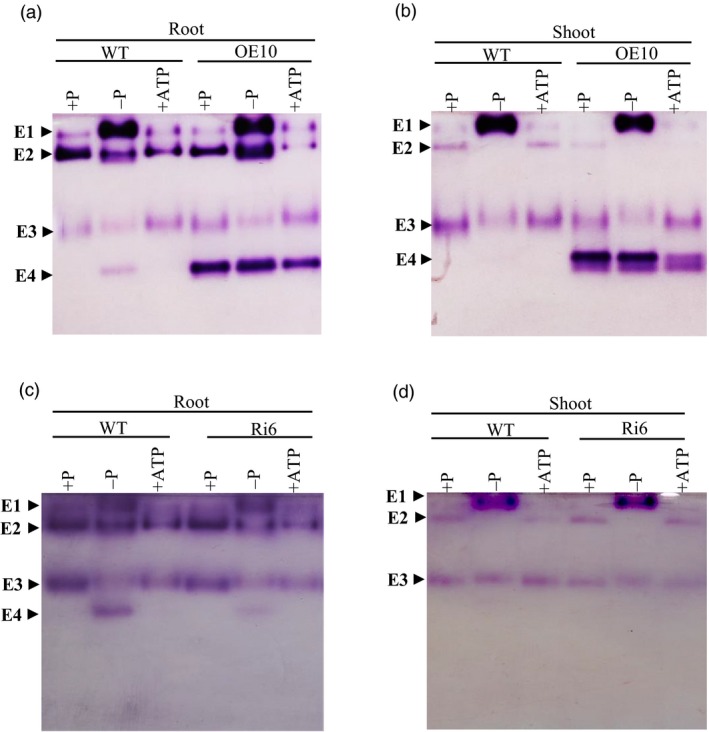
APase profiles of *OsPAP21b* transgenics and WT. (a) APase profiles of total root and (b) shoot proteins in 30‐day‐old WT and OE10 seedlings grown under +P, −P and +ATP conditions. (c) APase profiles of total root and (d) shoot proteins in 30‐day‐old WT and Ri6 under +P, −P and +ATP conditions. 10 μg of total root and shoot proteins were separated on 10% non‐reducing SDS‐PAGE. Gels were stained with fast black potassium salt and β‐naphthyl acid phosphate. Different APase isoforms E1, E2, E3 and E4 are indicated by arrow heads.

To investigate the effect of *OsPAP21b* overexpression on APase profiles, we performed in‐gel APase assays with total root and shoot proteins of WT and OE lines (Figure 8). Three isoforms could be clearly identified and were named as E1, E2 and E3. Similar isoforms were also identified earlier in rice by Tian *et al*. (2012). However, in our study a fourth isoform, named as E4 was spotted in WT root only under −P condition. This suggests that E4 is a low Pi‐inducible isoform that is predominantly induced in root tissues. Interestingly, intense overexpression of E4 isoform was observed in all OE lines as compared to WT, irrespective of treatment and tissue indicating E4 is indeed encoded by *OsPAP21b* (Figure 8a, b). Further, coomassie‐stained protein bands (from another non‐reducing PAGE gel) corresponding to E4 isoform were identified as OsPAP21b by mass spectrometry with a significantly high Mascot score (128) (Figure S11). Finally, reduction in this form in RNAi lines as compared to WT further validates its identity (Figure 8c, d). Taken together, these results confirm that OsPAP21b encodes a major low Pi‐inducible isoform in root.

### Effects of *OsPAP21b* silencing on plant growth

**Figure 9:**
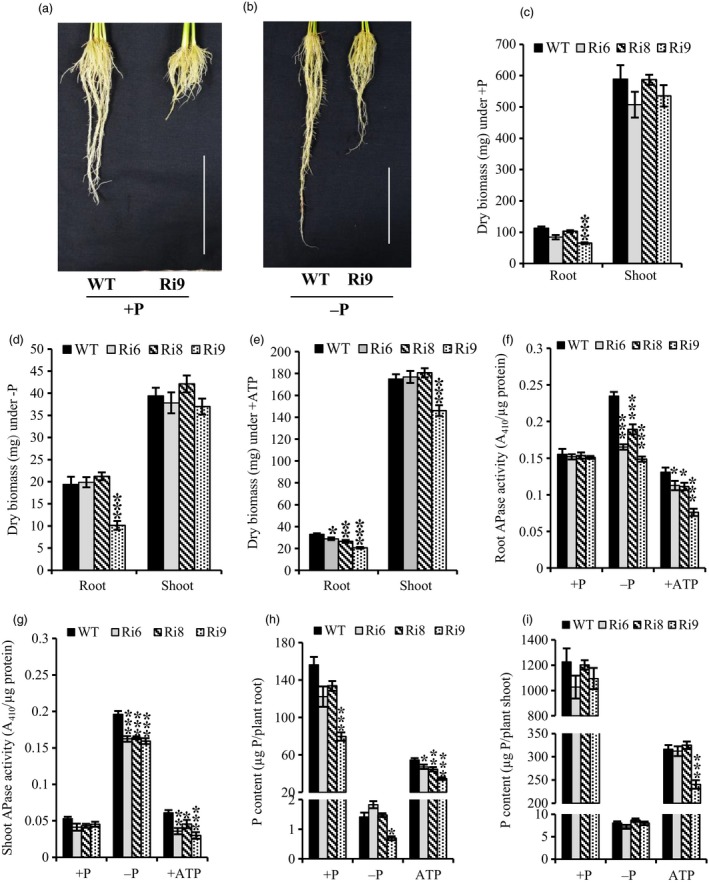
Analysis of RNAi lines of *OsPAP21b* for root phenotype, biomass, APase activity and P content. (a) Root growth of 30‐day‐old WT and Ri9 under +P and (b) −P (*n* = 5). Scale bar = 10 cm. (c–e) Root and shoot dry biomass under +P, −P and ATP recovery conditions. (f–g) Total APase activity and (h–i) total P content in roots and shoots of seedlings raised under +P, −P and +ATP conditions. Each value represents mean of three replicates with standard error. *P value <0.05; **P value <0.01; ***P value <0.001.

Realizing the positive influence of *OsPAP21b* overexpression on low Pi tolerance, we raised RNAi lines (Ri) of *OsPAP21b* to appraise its contribution under Pi deficiency. Similar to OE lines, Ri lines were also raised under +P, −P and +ATP conditions. Expression analysis of Ri lines revealed significant down‐regulation of *OsPAP21b* in all three conditions (Figure S12). Morphological analysis of Ri lines revealed significant decrease in root biomass in relation to WT under ATP recovery (Figure 9e). Notably, Ri9 with highest down‐regulation of *OsPAP21b* showed reduction in root biomass under all conditions as compared to WT (Figure 9c–e). However, no significant differences in shoot biomass were evident between Ri lines and WT under any treatments except for Ri9 which showed ~20% reduction in shoot biomass under +ATP (Figure 9e). Further, significant differences in root length were also observed only in Ri9 line as compared to WT under all three conditions, indicating significant role of *OsPAP21b* in affecting root architecture (Figure S13).

#### Silencing of OsPAP21b decreased APase activity and P content

Significant decrease in APase activity was observed in roots and shoots of RNAi lines grown in −P and ATP‐supplemented media as compared to WT (Figure 9f, g). Notably, about 20%–35% decrease in APase activity was observed under −P in roots of RNAi lines. This suggests significant contribution of OsPAP21b in total plant APase activity under −P. Further, quantitation of total P content of Ri lines revealed significant decrease in P content in roots under +ATP (Figure 9h). However, except for Ri9, no significant differences in root P content were observed in Ri lines under −P and +P conditions. Notably, any significant differences in shoot P content could not be observed between WT and Ri lines under any treatment except for Ri9 which showed significant reduction under +ATP (Figure 9i). Collectively, these results suggest the importance of OsPAP21b in hydrolysis of organic‐P compounds and improving Pi utilization in rice.

## Discussion

Organophosphates constitute ~80% of total soil P; however, remain unavailable for root uptake before mineralization. PAPs, especially secretory ones, are emerging as major plant enzymes for releasing Pi from these sources. Twenty‐six rice PAPs are classified into three main groups (I, II and III) and seven subgroups (Ia, Ib, Ic, IIa, IIb, IIIa, IIIb) (Zhang *et al*., 2011). Despite being a large gene family, only one PAP of subgroup Ic (OsPAP23) and two PAPs of subgroup Ia (OsPAP10a and OsPAP10c) have been shown to enhance the extracellular Pi utilization from organic‐P sources (Li *et al*., 2012b; Lu *et al*., 2016; Tian *et al*., 2012). Interestingly, both rice and Arabidopsis PAPs of subgroup Ic are reported to possess phytase activity (Li *et al*., 2012b; Wang *et al*., 2009; Zhang *et al*., 2011), whereas PAPs from subgroup Ia are major secretory PAPs in both rice and Arabidopsis (Lu *et al*., 2016; Tian *et al*., 2012; Tran *et al*., 2010b; Wang *et al*., 2011). Here, we have provided detailed characterization of one of the major low Pi‐responsive rice PAPs, *OsPAP21b* that belongs to a different subgroup, Ib which phylogenetically lies between Ia and Ic.

Several transcriptome studies have commonly reported a high induction of *OsPAP21b* under Pi deficiency across diverse rice genotypes suggesting its important role(s) in low Pi response (Li *et al*., 2010; Mehra *et al*., 2016; Pariasca‐Tanaka *et al*., 2009; Secco *et al*., 2013; Takehisa *et al*., 2013; Zhang *et al*., 2011). Notably, majority of these studies were conducted in roots. We also found preferential induction of *OsPAP21b* in roots which might be due to the active roles of roots in soil P solubilization and acquisition. Additionally, magnitude of *OsPAP21b* up‐regulation was progressively enhanced with prolonged exposure to Pi deficiency. These evidences indicate spatio‐temporal regulation of *OsPAP21b* in response to low Pi. Further, several PSR genes are positively controlled by MYB transcription factor, OsPHR2 during Pi starvation (Zhou *et al*., 2008). Earlier study by Zhang *et al*. (2011) indicated the role of OsPHR2 in regulation of PAPs by showing increased accumulation of *OsPAP21b* and other PAPs transcripts in *OsPHR2* overexpression lines. Our study confirmed that OsPHR2 indeed binds with the promoter of *OsPAP21b* and regulates its expression.

Several lines of evidences indicate a strict Pi status‐dependent regulation of OsPAP21b at both transcription and translational levels, and therefore, its key role in Pi acquisition and utilization. These are: (i) suppression of *OsPAP21b* accumulation in Pi‐starved WT within 1 h of Pi resupply in roots. (ii) Resupply of ATP to Pi‐starved *OsPAP21b* constitutive OE lines repressed both accumulation of *OsPAP21b* transcripts and total APase activity as compared to −P grown seedlings. Notably, suppression of *OsPAP21b* under Pi recovery was accompanied with simultaneous increased P accumulation in seedlings. (iii) Pi deficiency further enhanced the accumulation of *OsPAP21b* transcripts and protein in OE lines. This can be attributed to the overexpression of full‐length cDNA which contains full 5′ and 3′ UTRs (Figure S2). 5′ UTR is known to possess regulatory elements that can regulate gene expression. Further, the presence of a long 3′ UTR can potentially affect the stability of *OsPAP21b* transcripts in OE lines in response to Pi status. Such post‐transcriptional regulations of PAPs have also been reported for AtPAP10 (Wang *et al*., 2011). Similarly, AtPAP10 overexpression transgenics also showed increased PAP protein accumulation under −P condition despite having constitutive promoter (Zhang *et al*., 2014). (iv) In‐gel APase assays also confirmed increase and subsequent decrease in activity of E4 isoform (corresponding to OsPAP21b) under −P and ATP recovery, respectively. (v) Lastly, enzyme activity assays with recombinant OsPAP21b revealed complete inhibition of APase activity at a concentration of Pi (10 mm) which corresponds to cellular Pi levels. All these points indicate some post‐transcriptional or post‐translational regulation, which influences levels of either constitutively expressing *OsPAP21b* transcripts or protein in response to elevated Pi status. Such direct link between Pi status and OsPAP21b levels clearly proved its importance in Pi acquisition and utilization.

Plant microRNAs and Pi resupply‐inducible serine proteases have been proposed for such regulation (reviewed in Tran *et al*., 2010a). However, any direct evidences for these mechanisms are largely missing and needs further investigations. Interestingly, native western blotting of *OsPAP21b* OE lines with anti‐OsPAP21b antibody showed high molecular weight oligomer formations by OsPAP21b (Figure S14). Further, immunoblotting of non‐reducing SDS‐PAGE gel of total protein from OE10 also showed complexes of OsPAP21b. Interestingly, most of these complexes perfectly aligned with E1, E2 and E4 APase isoforms (Figure S15). Y2H assays (Figure S16); however, excluded any probable oligomerization of OsPAP21b protein with itself as suggested for several high molecular weight (HMWs ~55 kDa) PAPs (Li *et al*., 2002) or other co‐expressed PAPs. It seems that these putative OsPAP21b‐associated proteins may govern its protein level regulations. Moreover, transcriptional regulation of PAPs via PHR2 has also been proposed to be influenced by SPX proteins (Zhang *et al*., 2011). However, more in‐depth investigations of such regulations need to be carried out in future. Furthermore, while *OsPAP21b* transcript is repressed by Pi; however, even 48 h of Phi treatment did not have any significant effect on *OsPAP21b* expression. This indicates systemic regulation of *OsPAP21b*. Recently, Jost *et al*. (2015) also showed that transcripts of *AtPAP17* and *AtPAP1* are non‐responsive to Phi treatments which suggest that these PAPs could discriminate well between Pi and Phi.

Although overexpression of *OsPAP21b* led to increased protein accumulation and hence increased APase activity under +P conditions in OE lines as compared to WT, no significant or negative effect on plant growth performance under +P conditions was observed in OE lines. This could be due to no significant change in plant P content between WT and OE transgenics under +P conditions. Previous studies with *AtPAP10* also showed increased root‐associated APase activity of OE lines; however, no significant differences were detected between WT and transgenics phenotypically under +P conditions (Wang *et al*., 2011). Under −P conditions, OE lines showed significant increase in APase activity and P content of roots as compared to WT which suggests that OsPAP21b can also hydrolyse the intracellular organophosphates. Analysis of Ri lines and in‐gel APase isoforms also confirmed major contribution of OsPAP21b in total APase activity under −P. Down‐regulation of *OsPAP21b* in Ri9 led to a decrease in APase activity leading to decrease in P content and root biomass as compared to WT under −P. Noticeably, the most important role of OsPAP21b was observed in extracellular Pi utilization from different organic sources. Increased intracellular and secretory APase activity in OE lines sufficiently enhanced plant P content upon recovery with ATP which ultimately led to faster recovery of Pi‐starved OE lines as compared to WT. Unlike OE lines, Ri lines of *OsPAP21b* showed decreased APase activity and loss of root biomass under recovery conditions as compared to WT. Down‐regulation of *OsPAP21b* also led to loss of shoot biomass in Ri under ATP recovery indicating its major role in extracellular ATP utilization.

Notably, nucleic acids form a large portion of organic‐P in soil and need to be hydrolysed by phosphatases before root uptake (Turner, 2008). Additionally, it has been reported that plant cells secrete ATP in extracellular matrix to maintain cell viability (Chivasa *et al*., 2005). These secreted ATPs could also act as source of organic‐P for OsPAP21b. Apart from ATP, Pi‐starved OE lines also showed improved recovery on a variety of other organic‐P substrates. APase assay of recombinant OsPAP21b protein also confirmed its broad substrate specificity for variety of organophosphates. Further, OE lines of *OsPAP21b* also showed better growth as compared to WT in soil containing manure as organic‐P source. Taken all together, OsPAP21b with both intra‐ and extracellular APase activities proved to be an ideal candidate for improving low Pi tolerance in rice. Interestingly, none of the PAPs of Ib or any other subgroups other than Ia were earlier shown to be of secretory in nature. Identification and characterization of OsPAP21b also upholds the future possibility of identifying more potent secretory PAPs in rice. Nevertheless, moderate thermostability and acidic range pH optima of OsPAP21b makes it a suitable candidate for high‐temperature rice ecosystems especially low Pi acidic soils.

Our results further revealed a potential role of rice PAPs in modulating root system architecture under low Pi. Studies with *AtPAP10* and *NtPAP12* have also indicated role of PAPs in modulating RSA by hydrolysing cell wall bound enzymes involved in cell wall biosynthesis (Kaida *et al*., 2010; Wang *et al*., 2011). Arabidopsis mutants of several PAPs also show poor root growth as compared to WT (Wang *et al*., 2014). As several metabolic intermediates or signalling molecules are also substrate of OsPAP21b, an indirect role of OsPAP21b in controlling RSA is quite possible.

Rice, the staple food crop for more than half of the world's population, has only ~25% P use efficiency (Vinod and Heuer, 2012). Therefore, developing rice genotypes for improved P use efficiency has been recognized as a critical step towards its sustainable production. The present work is an extension of such efforts and identifies a novel PAP, OsPAP21b that can substantially enhance the solubilization and scavenging of abundant natural organic‐P sources. Further, Pi fertilizer loss to waterbodies poses great threat to aquatic life due to eutrophication. Such studies would also help to reduce the use of Pi fertilizers and protect environment.

## Experimental procedures

### Plant material and growth conditions

For hydroponic experiments, rice (*Oryza sativa* cv. PB1) seeds were surface‐sterilized and germinated in the dark as described previously (Mehra *et al*., 2016). Evenly germinated seeds were placed on nylon mesh floating over Yoshida growth medium, pH 5.0–5.5. Experiments were carried out in green house at 30/28 °C (day/night) temperature, 70% relative humidity and 16/8‐h photoperiod. For Pi sufficient and deficient treatments, nutrient media containing 320 μm (+P) and 1 μm NaH_2_PO_4_ (−P) were supplied, respectively. Different nutrient deficiency conditions (−N, −P, −K, −Fe and −Zn) were created as described (Mehra and Giri, 2016).

For +Phi/Pi treatments, seedlings were raised hydroponically under +P and −P conditions for 15 days, after which −P raised plants were supplied with 320 μm of NaH_2_PO_4_ or 320 μm of Phi (Na_2_HPO_3_·5H_2_O) for different time intervals, and tissues were collected for P content and gene expression analyses. Relative expression levels of *OsPAP21b* under −P and after Phi/Pi recovery were calculated with respect to expression under +P condition.

*OsPAP21b* overexpression and RNAi lines were grown in −P or +P media for 15 days. Afterwards, half of the Pi‐starved seedlings were recovered with 320 μm ATP (Sigma) for another 15 days. Subsequently, all 1‐month‐old +P, −P and ATP recovered −P seedlings (+ATP) were phenotyped. For studying performance of *OsPAP21b* OE lines on different organic substrates, 15‐day‐old −P grown seedlings were recovered with 320 μm of different organic substrates (ATP, ADP, p‐Ser, phytate) for another 15 days. For analysis of growth in soil system, 15‐day‐old hydroponically raised −P seedlings were recovered in soil supplemented with only organic manure (2 sand: 1 soilrite: 1 vermiculite: 1 organic manure) as P source (43 ± 0.7 mg/kg total P). Plants were raised in five replicates in greenhouse till 2 months of age before harvesting. Unless stated otherwise, experiments were performed in three biological replicates (*n* = 10–15).

### Biochemical characterization of OsPAP21b

Coding region of *OsPAP21b* was cloned into *NdeI* and *EcoRI* sites of pET28a vector (Novagen) and transformed in *E. coli*, BL21(DE3)pLysS cells. Transformed cells were treated with 0.3 mm IPTG at 15 °C for 18 h to induce the expression of 6XHis‐tagged OsPAP21b. Recombinant OsPAP21b protein was isolated and purified by Ni^2+^‐affinity chromatography as reported earlier (Mehra and Giri, 2016). Activity assays were performed with 1.5 μg of recombinant OsPAP21b protein in 100 μL reaction mixture containing 50 mm sodium acetate buffer pH 5.0, 5 mm MgCl_2_ and 10 mm pNPP (*p*‐nitrophenol phosphate) as standard substrate. Reactions were incubated at 37 °C for 30 min, and released Pi was measured by yellow vanadomolybdate method (Kitson and Mellon, 1944) by adding 100 μL of vanadate–molybdate reagent. For measurement of enzyme activity with different P‐containing substrates, all substrates were used at final concentrations of 10 mm in the reaction describe above. For measurement of activity at different pH, 50 mm of sodium acetate (pH 4–6) and Tris‐maleate buffer (6.5–8.0) were used with pNPP and ADP as substrates. Optimum temperature was determined by incubating reactions at different temperatures in Veriti™ thermal cycler (Applied Biosystems). For determination of cofactors and inhibitors of OsPAP21b, 10 mm chloride salts of different cations and sodium salts of different anions were used in reaction mixture with 10 mm pNPP as substrate. For calculation of IC_50_, 0 to 25 mm of NaH_2_PO_4_ was incubated with 10 mm of pNPP as substrate. Kinetics constant, *K*_*m*_ and *V*_max_ were estimated from Lineweaver–Burk plot of enzyme activity at different concentrations of pNPP and ADP (0.1–100 mm).

### Vector construction and rice transformation

To generate *OsPAP21b*‐overexpressing (OE) rice transgenics, full‐length cDNA (Figure S2) was cloned in Gateway‐compatible binary vector pANIC6B (Mann *et al*., 2012) under the transcriptional control of maize ubiquitin promoter (*pZmUbi1*). For raising RNAi transgenics of *OsPAP21b* (Ri), 307‐bp region of *OsPAP21b* cDNA was amplified and cloned in pANIC8b vector (Mann *et al*., 2012) by Gateway Technology (Invitrogen). *Agrobacterium*‐mediated transformation of rice genotype, PB1 was carried out as described (Toki *et al*., 2006). Resultant transformants (T0) were confirmed for the presence of transgene by PCR with gene‐specific primers of hygromycin phosphotransferase (*hpt*; Table S1, Figure S3) and histochemical GUS assay (Jefferson *et al*., 1987). All experiments were subsequently performed with T3 generation homozygous transgenic lines. Overexpression and down‐regulation of *OsPAP21b* in transgenic lines was confirmed by qRT‐PCR using primers listed in Table S1.

For plant phenotyping, qRT‐PCR, EMSA, protein extraction, quantitation of total plant and secretory APase activity, in‐gel APase profiling and mass spectrometry, activity staining of root surface‐associated APases, yeast two‐hybrid assays, generation of anti‐OsPAP21b antibody, immunoblot analysis and total P content analysis, please see Supplementary methods.

## Conflict of interest

The authors declare no conflict of interest.

## Supporting information


**Figure S1** Induction and purification of recombinant OsPAP21b.
**Figure S2** Full‐length cDNA of *OsPAP21b*.
**Figure S3** Raising of rice transgenics with *OsPAP21b*.
**Figure S4** Effect of *OsPAP21b* overexpression on lateral root length.
**Figure S5** Root and shoot lengths of WT and *OsPAP21b* OE lines under (a) +P, (b) −P and (c) ATP recovery conditions.
**Figure S6** Growth performance of *OsPAP21b* OE lines on different organophosphates.
**Figure S7** Early flowering in *OsPAP21b* OE lines.
**Figure S8** Effect of *OsPAP21b* on expression of low Pi‐inducible PAPs.
**Figure S9** Effect of *OsPAP21b* on expression of low Pi‐inducible phosphate transporters (PTs).
**Figure S10** Co‐localization of YFP‐OsPAP21b with organelle markers.
**Figure S11** LC‐MS/MS identification of E4 APase isoform in *OsPAP21b* OE lines.
**Figure S12** Plant phenotype of *OsPAP21b* RNAi lines.
**Figure S13** Effect of *OsPAP21b* silencing on root and shoot lengths.
**Figure S14** OsPAP21b form high molecular weight complex in plant.
**Figure S15** Alignment of APase profile of OE10 with immunoblot of OE10 protein probed with anti‐OsPAP21b antibody.
**Figure S16** Yeast two‐hybrid assays of OsPAP21b with different PSR PAPs.
**Table S1** List of primers used in the study.
**Supporting methods** Plant phenotyping, qRT‐PCR, electrophoretic mobility shift assay (EMSA), total protein extraction and quantitation of APase activity in roots and shoots, detection of in‐gel APase profile and mass spectrometry of target protein, quantitation of secretory APase activity, activity staining of root surface‐associated APases, yeast two‐hybrid assays, generation of anti‐OsPAP21b antibody and immunoblotting of OsPAP21b and total P content analysis.
