# Supplementary material for: Improvement in phosphate acquisition and utilization by a secretory purple acid phosphatase (OsPAP21b) in rice
Source: Plant Biotechnol J. 2017 Mar 2;15(8):1054–67. doi: 10.1111/pbi.12699 (PMC5506657; doi:10.1111/pbi.12699)
Supplement: Supplementary file 1 — Figure S1 Induction and purification of recombinant OsPAP21b. Figure S2 Full‐length cDNA of OsPAP21b. Figure S3 Raising of rice transgenics with OsPAP21b. Figure S4 Effect of OsPAP21b overexpression on lateral root length. Figure S5 Root and shoot lengths of WT and OsPAP21b OE lines under (a) +P, (b) −P and (c) ATP recovery conditions. Figure S6 Growth performance of OsPAP21b OE lines on different organophosphates. Figure S7 Early flowering in OsPAP21b OE lines. Figure S8 Effect of OsPAP21b on expression of low Pi‐inducible PAPs. Figure S9 Effect of OsPAP21b on expression of low Pi‐inducible phosphate transporters (PTs). Figure S10 Co‐localization of YFP‐OsPAP21b with organelle markers. Figure S11 LC‐MS/MS identification of E4 APase isoform in OsPAP21b OE lines. Figure S12 Plant phenotype of OsPAP21b RNAi lines. Figure S13 Effect of OsPAP21b silencing on root and shoot lengths. Figure S14 OsPAP21b form high molecular weight complex in plant. Figure S15 Alignment of APase profile of OE10 with immunoblot of OE10 protein probed with anti‐OsPAP21b antibody. Figure S16 Yeast two‐hybrid assays of OsPAP21b with different PSR PAPs. Table S1 List of primers used in the study. Supporting methods Plant phenotyping, qRT‐PCR, electrophoretic mobility shift assay (EMSA), total protein extraction and quantitation of APase activity in roots and shoots, detection of in‐gel APase profile and mass spectrometry of target protein, quantitation of secretory APase activity, activity staining of root surface‐associated APases, yeast two‐hybrid assays, generation of anti‐OsPAP21b antibody and immunoblotting of OsPAP21b and total P content analysis. [file PBI-15-1054-s001.pdf]

Table S1 List of primers used in the study

	**Forward (5'-3')**	**Reverse (5'-3')**
OsPAP21b_pET28a/pGADT7/pGBKT7	GGAATTCCATATGGCGAGCTCGTGGAG	TGCTTAGAATTCCTACAGCTCGTCGGCC
OsPHR2_pET28a	GGAATTCCATATGGAGAGAATAAGCACCAAT	TGCTTAGGATCCTTATCTGTCACCTGATTCTG
OsPAP21b_pANIC6B	CACCGGGGTTGCGTTCCG	GGATGATAAGTATAAATAGTATAAG
OsPAP21b_pANIC8B	CACCAGCCAGCTGCTACAGT	GTATAAGCGAAAAGATGAGCCCATC
OsPAP21b_EMSA	AAAAATCCCTGCATCACGCAG	ACGAGGAAAACTACAGTTATCTGC
OsPAP21b_pSITE3CA	CACCATGGCGAGCTCGTGGAGGCT	CAGCTCGTCGGCCGGATGGCC
hpt	TCTACACAGCCATCGGTCCAG	GATGTAGGAGGGCGTGGATATG
OsPAP21b_qRT	GGCCCAATGTACATCACCATT	TGCCCCTTGATGAACTTGAGA
OsUBQ5_qRT	ACCACTTCGACCGCCACTACT	ACGCCTAAGCCTGCTGGTT
OsPAP1a_pGADT7	AGAATTCCATATGGCGGCGGCGGC	GCTTAGAATTCTCACGAGGCCAGGGTGGTGGT
OsPAP1d_pGADT7	AGAATTCCATATGATCAGGCTTTGGGTGGTGG	GATTAGGATCCTTAGGTAGCCAGTGTGGTCCGAGG
OsPAP3b_pGADT7	GGAATTCCATATGGCTGTTGCACTCGC	TGCTTAGAATTCTCATTCTTCAGTTACACTAGCAGA
OsPAP9b_pGADT7	GGAATTCCATATGGCGCGCCTCCTTC	TGCTTAGGATCCTCATCTGGCTCCCTTCA
OsPAP10a_pGADT7	CTTAGAATTCATGGTGGACCGGATCGGC	GATTAGGATCCTCACTGGGAGTCGTCCAAAGATTC
OsPAP10c_pGADT7	AATTCCATATGGGGATGCTGCGGTGG	GCTTAGAATTCTTATACATCGTCGTTGGTGGGCA
OsPAP27a_pGADT7	GCTTAGAATTCATGGCGATGCCGCTGGGTGG	ATTAGGATCCTCACGTCGCCGGCGGGATG
OsPAP3c_pGADT7	ATAATTCCATATGGCGAGGAGGAGCAG	GCTTAGAATTCTCATGCTTCAGTGATATAGGTGGA
OsPAP1a_qRT	CATTGCATCCAGTGTGCCTTA	TCCAGGCCAATCTCTTTCATG
OsPAP1d_qRT	TGTCGCAGTGGGATCAGTTC	GTATGGCACAGTCGAAGCAATC
OsPAP3b_qRT	GCTCGATTCGCCTTCTACGA	TTTGCTGAAGCTCCAGTGGT
OsPAP9b_qRT	CCTCCCTCTTCTGTGCCACTA	TAGCCCGGGTCGCTTGT
OsPAP10a_qRT	CGCCATCCTGGACATCAAG	TTGCGGTGCCACGTGTAG
OsPAP10c_qRT	GCCACGTCCACTCGTACGA	CGTTCACGATGTTGTAGTCGATGT
OsPAP23_qRT	TCGAACCACAGGGACAAGGT	GCGAATCTTGCCAGGTAGGAT
OsPAP27a_qRT	TGCTCGAGTCCCCTACATGAT	ATCCACTGTTTGGCCAATCC
OsPAP20b_qRT	CTACCACTCCGGCAACATCC	GTAGGTGGTGCTGGGTTTGA
OsPT1_qRT	CCTCGACATCGCCTTCTACAG	CACCCGACCTTGCTGAAGAT
OsPT2_qRT	GCACAAACTTCCTCGGTATGC	CAGCGACAAGCCCTTGGA
OsPT4_qRT	TCGGCGTGCGCAACT	GGCACCAGGAACGTGAAGAA
OsPT6_qRT	GCAATTAAGCTGGCATGGGC	GGTACCATTGCGTCTTGGC
OsPT8_qRT	GTCCCGGGCTACTGGTTCA	TGAAAAACCCTAGCAGCTGGAT
OsPT9_qRT	CCGGCTACATCGTGCTCTACT	AGGATGAAGGTGGTGGTGTTG

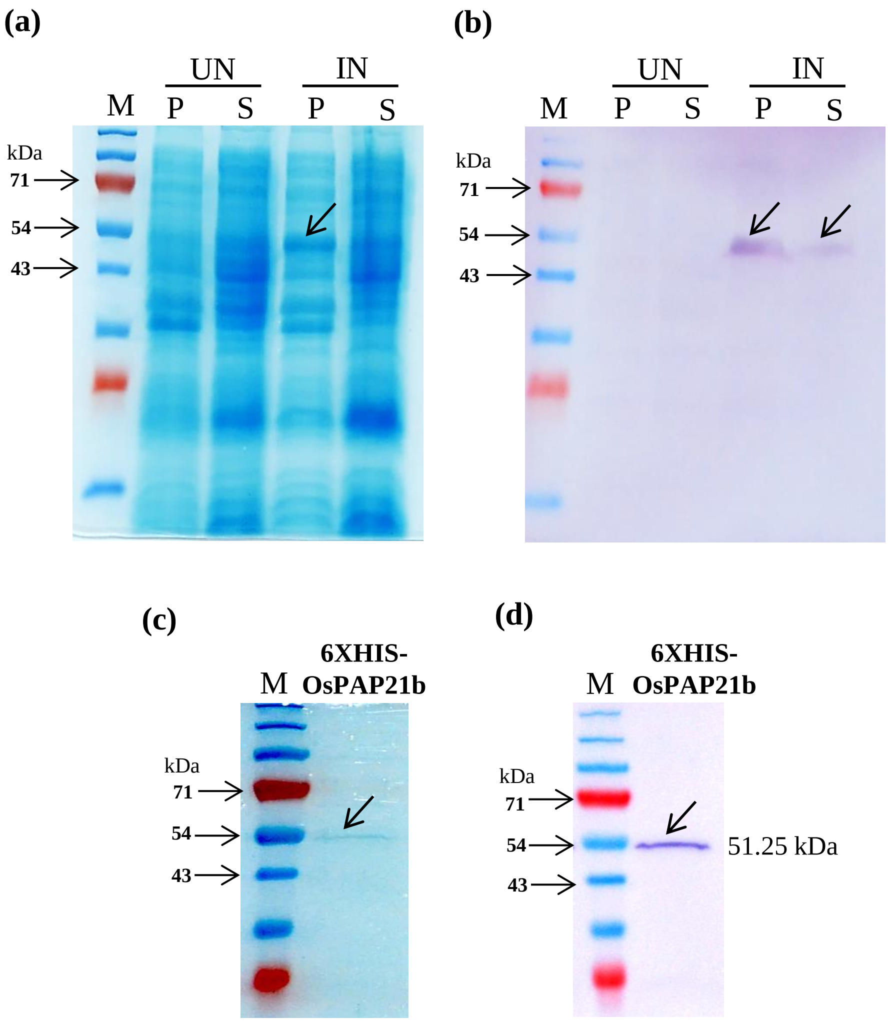

Figure S1. Induction and purification of recombinant OsPAP21b. (a) Overexpression of 6XHis-OsPAP21b in *E. coli* BL21(DE3) induced by IPTG. Induced (IN) and un-induced (UN) total protein fractions were separated on 12% SDS-PAGE gel. Gel was stained with coomassie blue. P and S indicate proteins in pellet (insoluble) and supernatant (soluble) fractions. 10 µg of protein was loaded in each lane. (b) Immunoblot of identical gel probed with anti-OsPAP21b antibody. (c) Coomassie-stained SDS-PAGE gel showing 51.25 kDa recombinant OsPAP21b protein (2 µg) purified by Ni^{2+}-affinity chromatography. (d) Western blot analysis of purified 6XHIS-OsPAP21b with anti-OsPAP21b antibody.

(a)

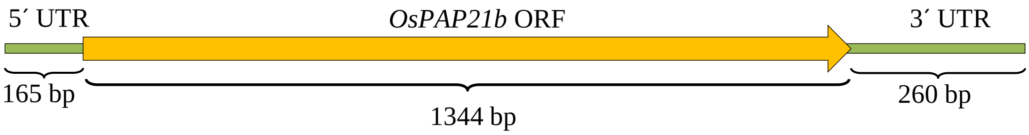

(b)

AK073222

GGGGTTGCGTTCCGCGTCGCCGTGGTTGCTCCGGCGAGAAGCCTCGTCGCCGTCGCGGGC
GCCGCCTTCTCCGCCGTGAACGCGCGGTGCGCGTGGTGCCTGGAACAGGCGGCGCTCGGG
CGGAGCTGCACGGGGACGGTCCTCGGCGACGCGGTGGTGGGCGCC ATG GCG AGC TCG
TGG AGG CTG CTG CTG CAG GGG ATC ACG TCG CTG GTG TTC CTG TGC GCG CGT GGC GCC
GAC GAG TAC GTC CGG CCG CCA CCG AGC CCG CTG GTG CTC ACG GCG CAC GGC AAG CCG
GCG TCG CAT CCT CAG CAG GTG CAC ATT TCG ATG GTG GGG GAG AAG AAC ATG AGG ATT
TCA TGG GTG ACC GAC GAC CTG AAC GCG CCG TCC GTG GTG GAG TAC GGG ACG TCG CCG
GGG AAG TAC ACG GCG TCG GCG ACC GGC GAC CAC ACC ACG TAC CGC TAC TTC CTC TAC
AAG TCC GGC GCG ATC CAC CAC GCC ACC ATC GGC CCG CTC GAG GCG AGC ACG ACG TAC
CAC TAC CGG TGC GGC AAG GCC GGC GAC GAG TTC ACC CTC AGG ACG CCT CCG GCG AGG
CTG CCT GTG GAG TTC GTC GTG GTC GGC GAC CTC GGG CAG ACG AAG TGG ACG GCG TCG
ACG CTG TCC CAC ATC GGC GGC GGC GGC GGC GAC TAC GAC GTG CTG CTG CTC CCC GGC
GAC CTG TCG TAC GCC GAC ACG CAG CAG CCG CTG TGG GAC ACG TTC GGG CGG CTG GTG
CAG CCG CTG GCG AGC GCG CGG CCG TGG ATG GTG ACG GAG GGG AAC CAC GAG ATC GAG
GCG CTG CCG GTG GTG GGG ATC GCG CCG TTC GCG GCG TAC AAC GCG CGG TGG CGG ATG
CCG CGG GAG GAG AGC GGC TCG CCG TCG AAC CTC TAC TAC TCG TTC GAC GCG GCG GGC
GGC GCG GCG CAC GTG GTG ATG CTG GGA TCC TAC GCG GAG TTC GAG GAA GGG TCG CCG
CAG CGG GCG TGG CTG GAG CGG GAC CTC GCC GGC GTC GAC CGG CGG CGG ACG CCG TGG
CTG CTG GCG CTG GTG CAC GCG CCG TGG TAC AAC ACG AAC GAG GCG CAC CAG GGC GAG
GGC GAG CGG ATG CGC CGC GCC ATG GAG AGC CTC CTC TAC GAG GCC CGC GTC GAC GTC
GTC TTC GCC GGC CAC GTC CAC GCC TAC GAG CGC TTC ACG AGG ATC TAC GAC AAC GAG
GCT GAC AGC CGG GGC CCA ATG TAC ATC ACC ATT GGC GAC GGG GGC AAC AGG GAA GGC
CTT GCT CTC AAG TTC ATC AAG GGG CAC AAG TCG GCG CAC CTG TCG GAG TTC AGG GAG
GCG AGC TTC GGG CAC GGG CGG CTG AGG GTC CTG AAC GAG ACG AGC GCC GTC TGG ACG
TGG CAC CGC AAC GAC GAC CAG TTC GCC ACC GTC CGC GAC GAG GTC TGG CTG CAC AGC
TTG GCC GCC GGC GAG CCA GCT GCT ACA GTA GCC TCC GCC GCC GGC GGC GGC GGC GGC
CAT CCG GCC GAC GAG CTG TAG **GCTATATATATCTCACACTGTTAAGAATGAATGGAAGAA**
AAGGAAACTTCACCACATTTAGCATTGTCTTTTATTTTTTTTCTTTTTCATCAGCCATAAACA
TTGTCCATTTGTCCTTGTAAATCAATCGTGATATCGTGTGTTTCAGCTAGTGTACAGTGTTTT
TCTTGTGTTAGACACGTGGTGAAATAGAATTTCAGTGTACAAGGCCAACACTGATGGGCTCA
TCTTTTCGCTTATACTATTTATACTTATCATCC

Figure S2. Full length cDNA of *OsPAP21b*. (a) Schematic representation of full length cDNA (1769 bp) of *OsPAP21b* overexpressed in rice. (b) Nucleotide sequence of full length cDNA of *OsPAP21b* with 5′UTR, CDS (red font) and 3′UTR.

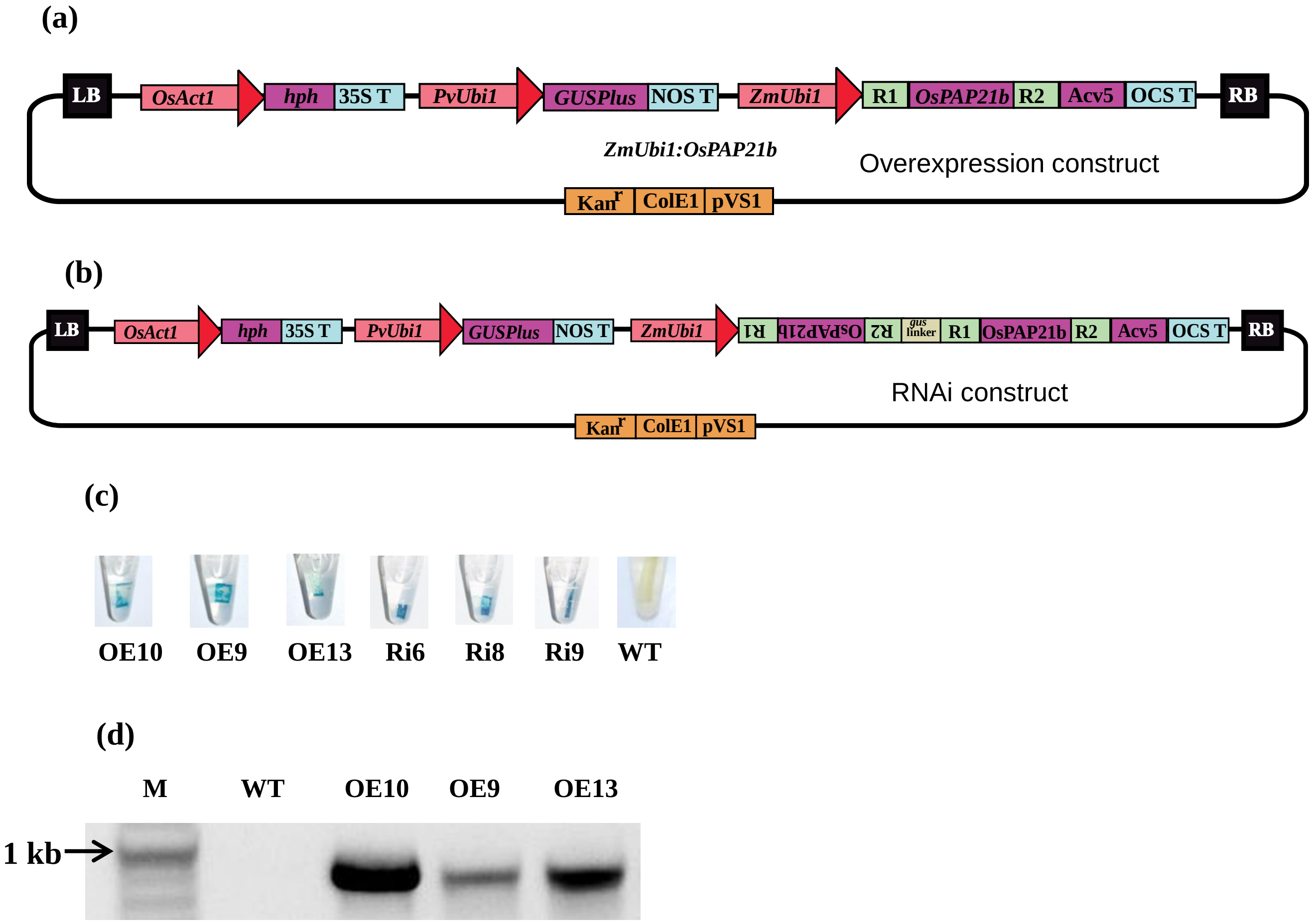

Figure S3. Raising of rice transgenics with *OsPAP21b*. (a) Schematic of *OsPAP21b* overexpression construct in Gateway-compatible destination vector, pANIC6B. *ZmUbi1* represents maize *Ubiquitin* promoter. (b) Schematic of *OsPAP21b* silencing construct in Gateway-compatible destination vector, pANIC8B. (c) Screening of rice transgenic lines by GUS histochemical assay. Leaf of plants showing positive GUS staining represents confirmed transgenics. (d) Screening of rice transgenic lines by PCR with gene-specific primers of hygromycin phosphotransferase (*hpt*).

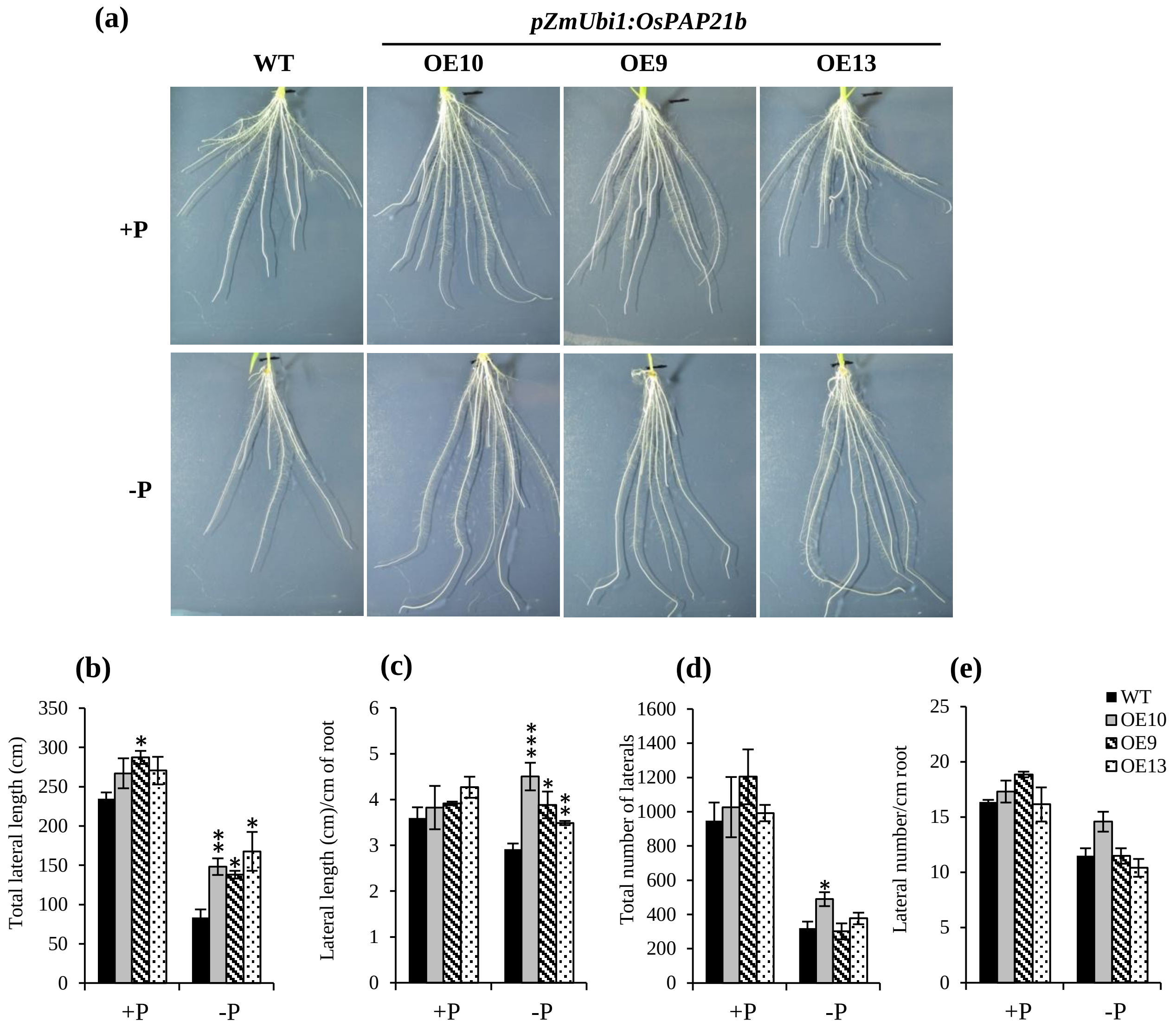

Figure S4. Effect of *OsPAP21b* overexpression on lateral root length. (a) Root of 12-days-old WT and *OsPAP21b* OE lines. Black bar at the top of each image represents scale of 1 cm. (b) Total lateral length per root of WT and OE lines. Total lateral length was determined by summing up lengths of all lateral roots that were produced in whole one root of each plant. (c) Total lateral length per cm of root in WT and OE lines. Total lateral length in whole one root was divided by total root length of non-lateral roots. (d) Total number of laterals per root of WT and OE lines. (e) Lateral number per cm of root of WT and OE lines. Total number of laterals was divided by total root length of non-lateral roots in each plant. Each value represents mean of three replicates with standard error. Level of significance was analysed with student's *t*-test. * *p* value < 0.05; ** *p* value < 0.01; *** *p* value < 0.001.

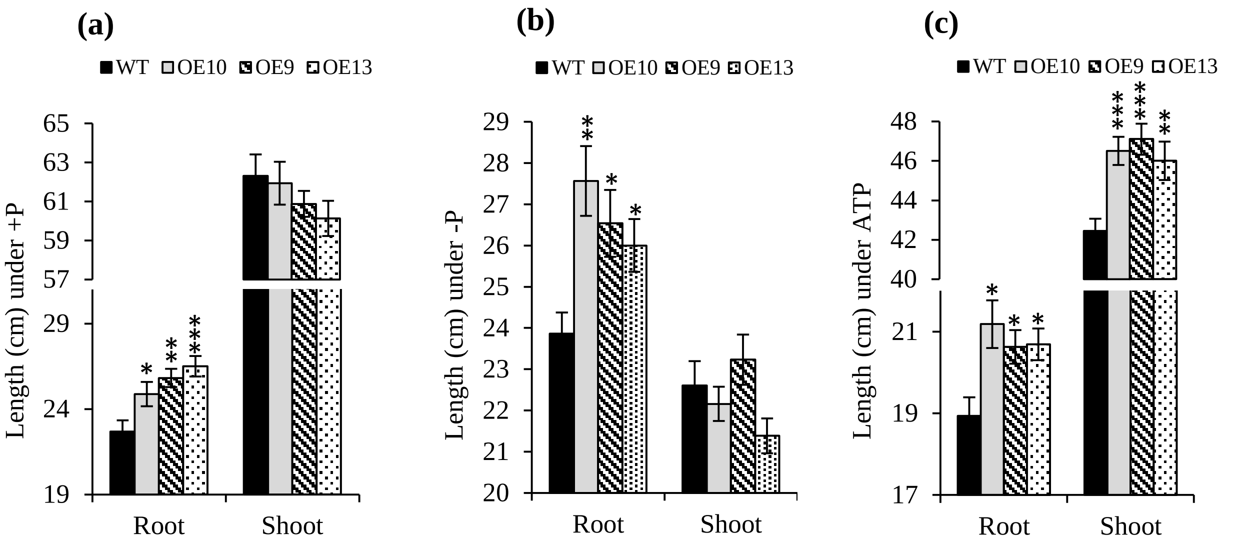

Figure S5. Root and shoot lengths of WT and *OsPAP21b* OE lines under (a) +P, (b) -P and (c) ATP recovery conditions. Plants were raised hydroponically for 30 days under +P, -P and +ATP recovery conditions (15 days Pi-starved seedlings were recovered with ATP for 15 subsequent days). Each values represents mean of 15 seedlings with standard error. Level of significance was analysed with student's *t*-test. * p value < 0.05; ** p value < 0.01; *** p value < 0.001.

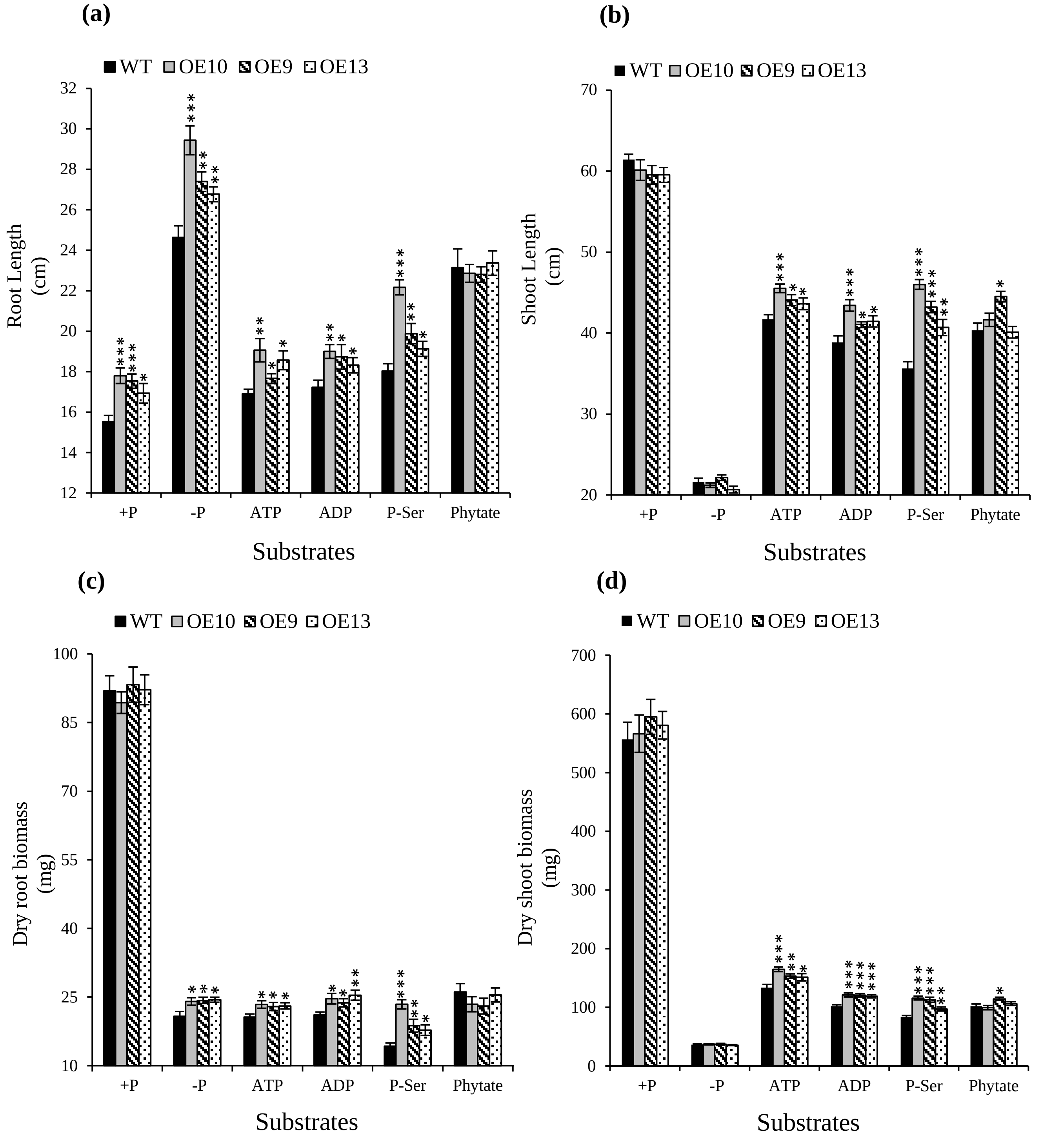

Figure S6. Growth performance of *OsPAP21b* OE lines on different organophosphates. (a) Root length, (b) shoot length, (c) dry root and (d) shoot biomass of WT and OE lines under +P, -P and after recovery of P-starved seedlings with different P containing organic substances. Plants were raised hydroponically for 30 days under +P, -P and recovery conditions (15 days Pi-starved seedlings were recovered with organophosphates for 15 subsequent days). ATP, ADP, phosphor-serine and phytic acid were used for recovery of Pi-starved seedlings. Each values represents mean of 15 seedlings with standard error. Level of significance was analysed with student's *t*-test. * p value < 0.05; ** p value < 0.01; *** p value < 0.001.

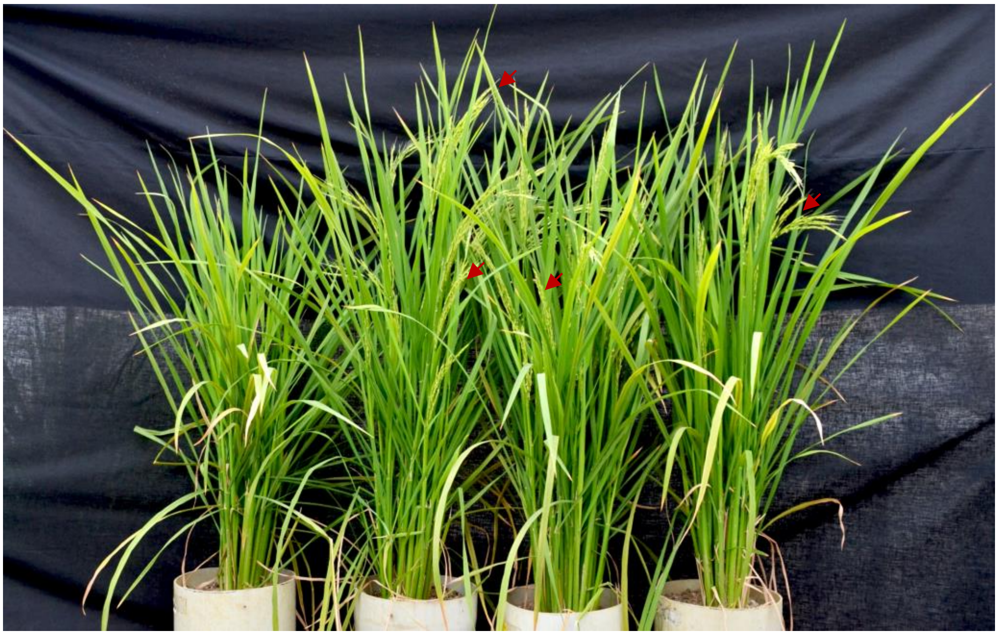

WT **OE10** **OE9** **OE13**

Figure S7. Early flowering in *OsPAP21b* OE lines. Early panicle emergence (few indicated by arrow heads) in overexpression lines of *OsPAP21b* as compared to WT in soil system containing organic manure as sole P source.

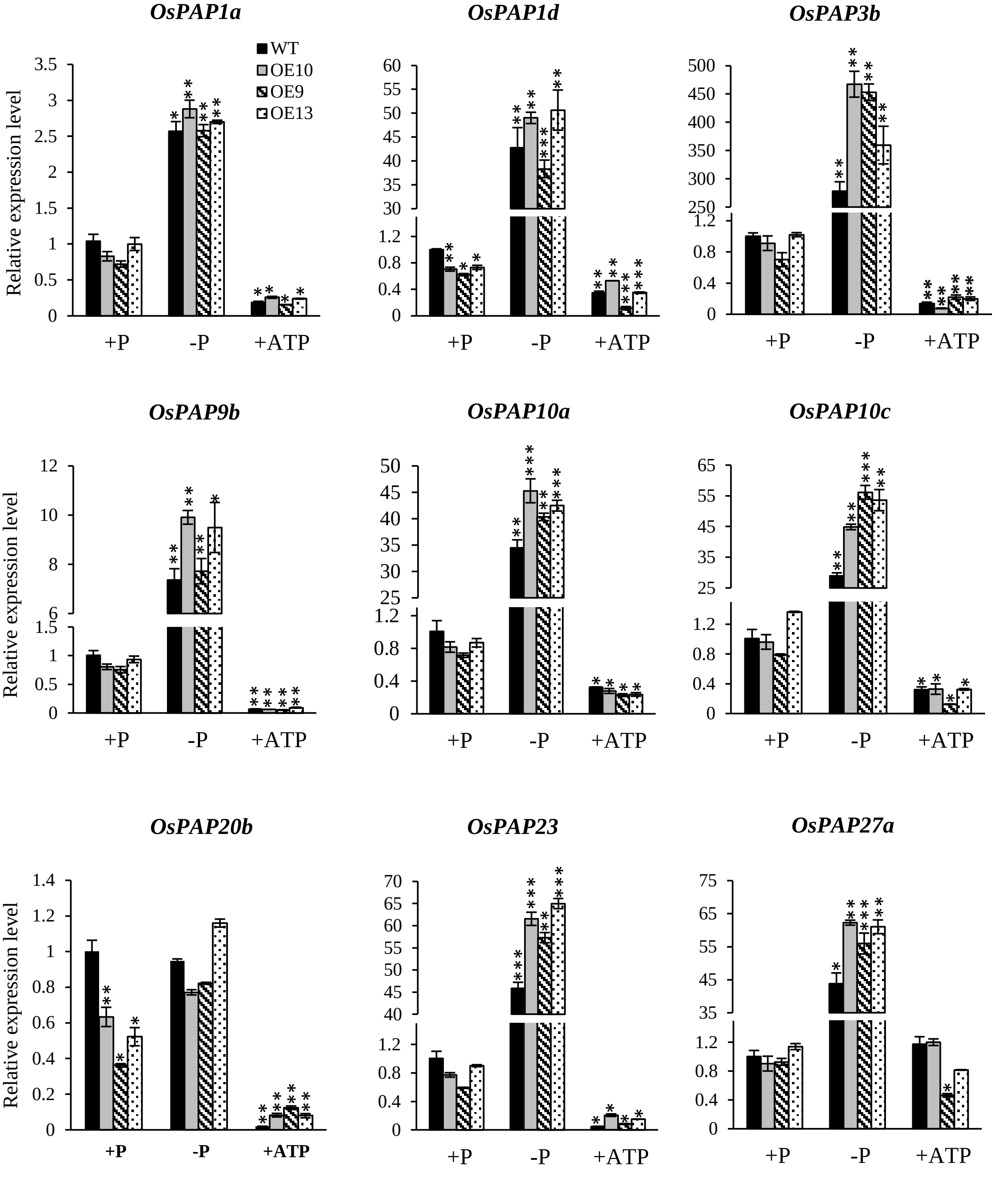

Figure S8. Effect of *OsPAP21b* on expression of low Pi inducible PAPs. Expression profiling of *OsPAP*s in roots of WT and OE lines of *OsPAP21b* by qRT-PCR under +P, -P and +ATP. Relative expression and level of significance were determined with respect to expression levels in WT under +P conditions. Each value represents mean of three biological with standard error. Level of significance was analysed with student's *t*-test. * *p* value < 0.05; ** *p* value < 0.01; *** *p* value < 0.001.

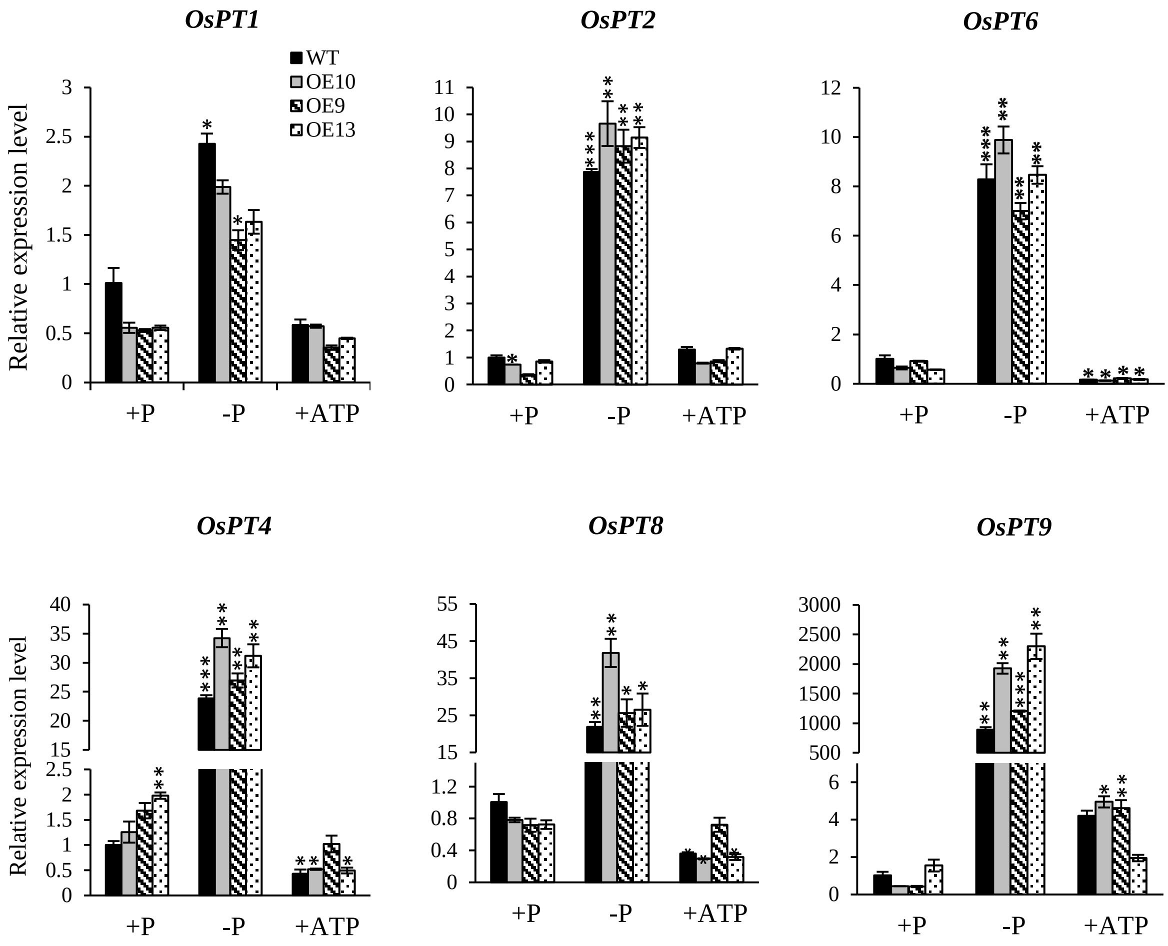

Figure S9. Effect of *OsPAP21b* on expression of low Pi inducible phosphate transporters (PTs). Expression profiling of *OsPT*s in roots of WT and OE lines of *OsPAP21b* by qRT-PCR under +P, -P and +ATP. Relative expression and level of significance were determined with respect to expression levels in WT under +P conditions. Each value represents mean of three biological replicates with standard error. Level of significance was analysed with student's *t*-test. * p value < 0.05; ** p value < 0.01; *** p value < 0.001.

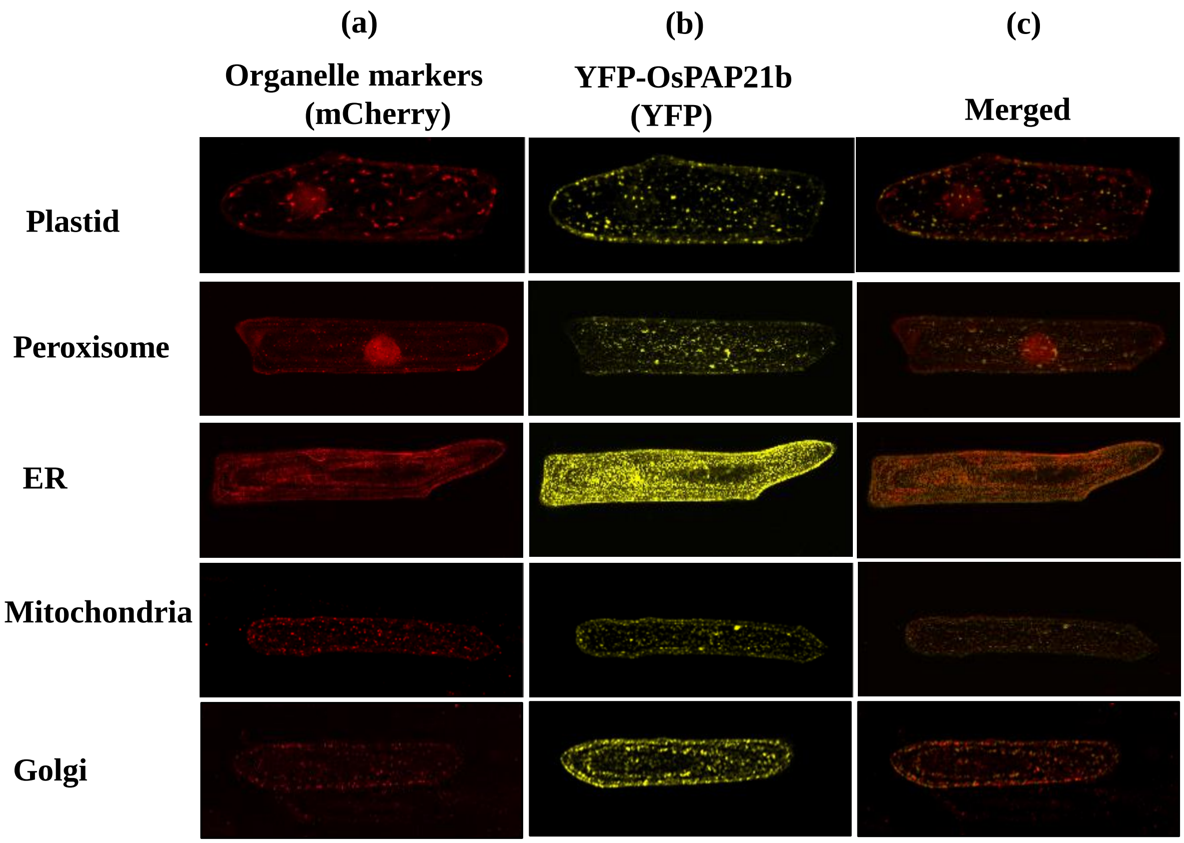

Figure S10. Co-localisation of YFP-OsPAP21b with organelle markers. (a) Transient expression (red fluorescence) of organelle markers fused with mCherry in onion peels. Different organelle markers used for co-localisation; plastid (PT-rk, ABRC stock: CD3-999), peroxisome (PX-rk, ABRC stock: CD3-983), endoplasmic reticulum (ER-rk, ABRC stock: CD3-959), mitochondria (MT-rk, ABRC stock: CD3-991) and golgi bodies (G-rk, ABRC stock: CD3-967). (b) Transient expression of OsPAP21b fused with YFP (yellow signals) in onion epidermal cells. (c) Merged images showing superimposition of images in (a) and (b) panels. Images were captured using AOBS TCS-SP2 confocal microscope.

(a)

gi|62732718 **Mass:** 59880 **Score:** 128 **Matches:** 3(2) **Sequences:** 3(2) **emPAI:** 0.14
Ser/Thr protein phosphatase family protein, putative [Oryza sativa Japonica Group]

1 MMMATAAMAA SSCDRGDTRK KLQITVVFLV RTLLLACIIA RGVLALIRVA
51 FRVAVVAPAR SLVAVAGAAF SAVNARCAWC LEQAALGRSC TGTVLGDAVV
101 GAMASSWRLL LQGITSLVFL CARGADEYVR PPPSPLVLTA HGKPASHPQQ
151 VHISMVGEKN MRISWVTDDL NAPSVVEYGT SPGKYTASAT GDHTTYRYFL
201 YKSGAIHHAT IGPLEASTTY HYRCGKAGDE FTLRTPPAR**L** **PVEFVVVGDL**
251 **GQTK**WTASTL SHIGGGGGDY DVLLLPGDLS YADTQQPLWD TFGRLVQPLA
301 SARPWMVTEG NHEIEALPVV GIAPFAAYNA RWRMPREESG SPSNLYYSFD
351 AAGGAAHVVM LGSYAEFEEG SPQRAWLERD LAGVDRRRTP WLLALVHAPW
401 YNTNEAHQGE GERMRR**AMES** **LLYEAR**VDVV FAGHVHAYER FTRIYDNEAD
451 SR**GPMYITIG** **DGGNR**EGLAL KFIKGHKSAH LSEFREASFG HGRLRVLNET
501 SAVWTWHRND DQFATVRDEV WLHSLAAGEP AATVASAAGG GGGHPADEL

(b)

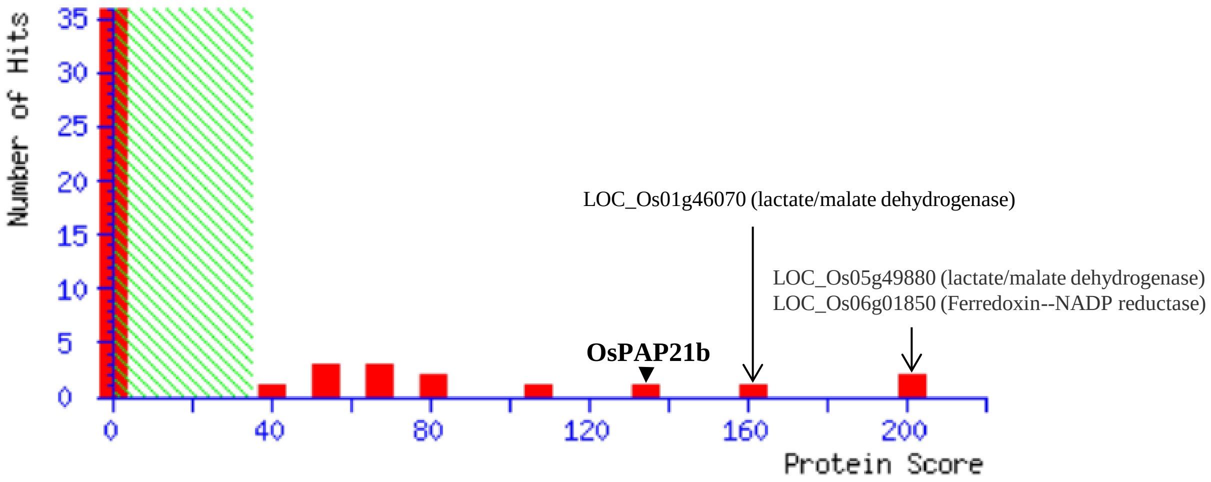

Figure S11. LC-MS/MS identification of E4 APase isoform in *OsPAP21b* OE lines. (a) Snapshots of Mascot search result window showing identification of OsPAP21b in E4 isoform. Matched peptides in OsPAP21b protein sequence are highlighted in red bold letters. (b) Mascot score histogram showing identification of OsPAP21b (indicated by arrow head) with a high Mascot score of 128 at $p < 0.05$. Peptide spectra were analysed and identified using Analyst QS Software (version 2.0) and Mascot search engine (version 2.1), respectively.

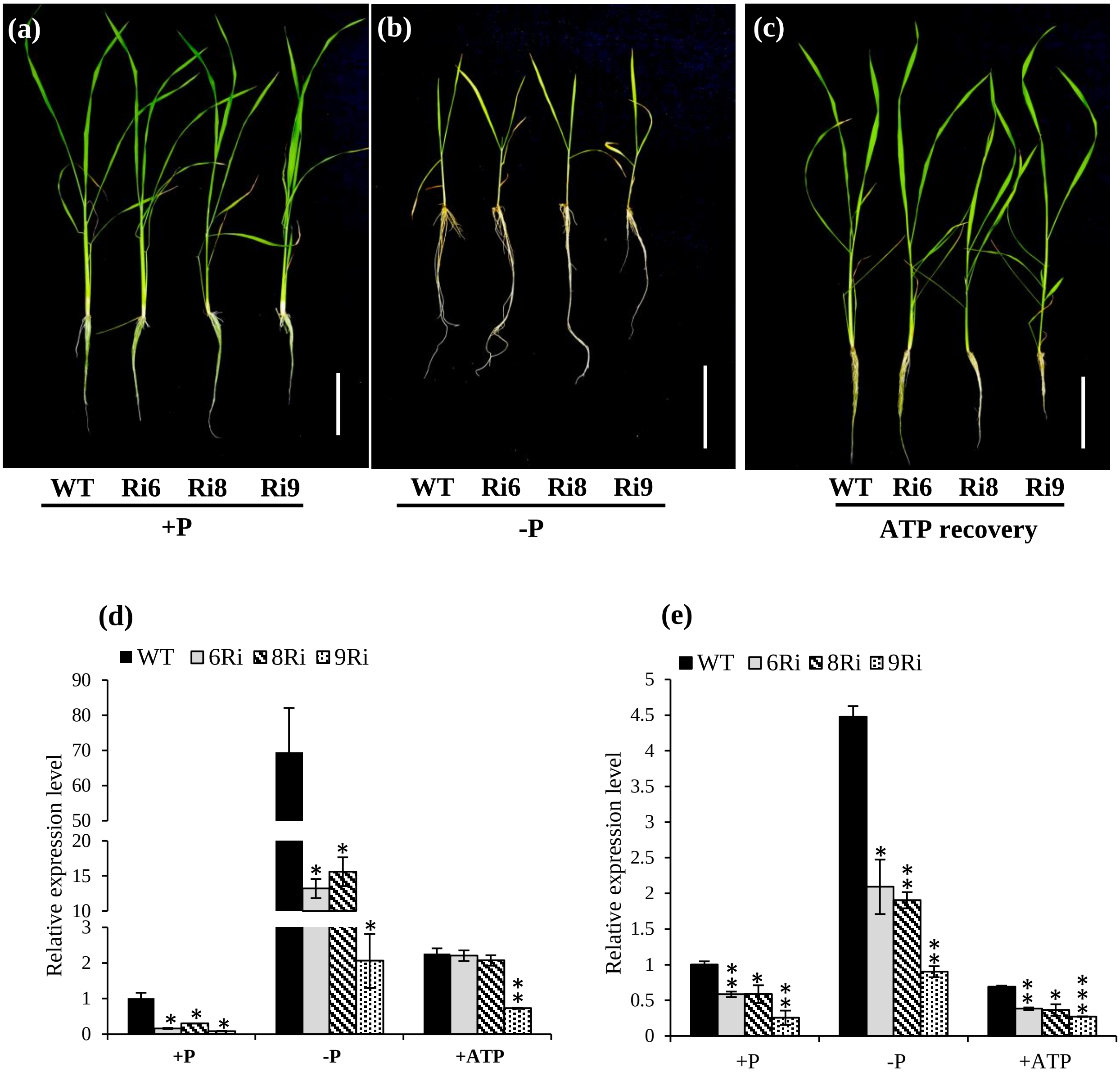

Figure S12. Plant phenotype of *OsPAP21b* RNAi lines. (a-c) Phenotype of 30-days-old WT and *OsPAP21b* RNAi lines under +P, -P and +ATP conditions. Scale bar = 10cm. (d) Relative expression levels of *OsPAP21b* in RNAi lines in roots and (e) shoots. Relative expression and level of significance were determined with respect to expression levels in WT under +P conditions. Each value represents mean of three biological replicates with standard error. Level of significance was analysed with student's *t*-test. * p value < 0.05; ** p value < 0.01; *** p value < 0.001.

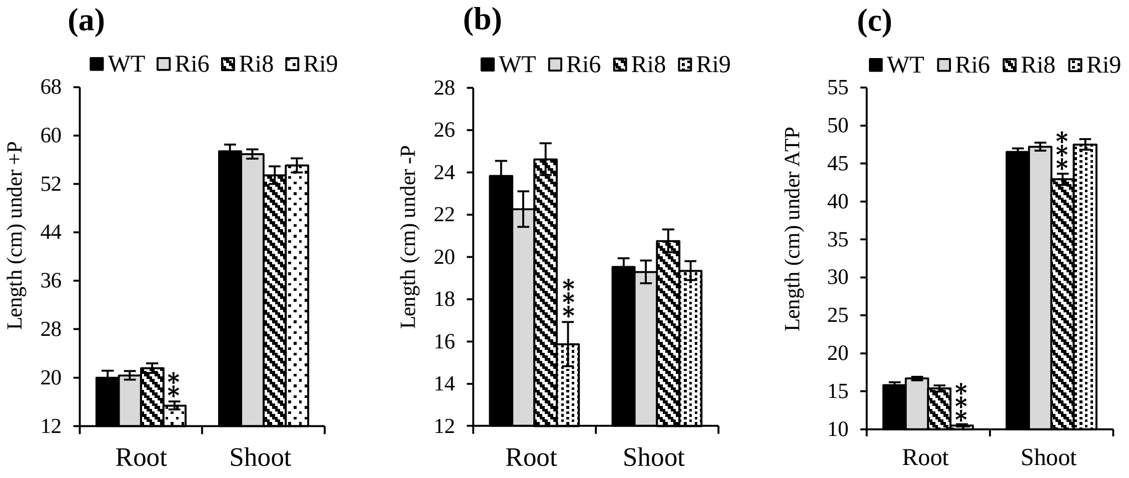

Figure S13. Effect of *OsPAP21b* silencing on root and shoot lengths. (a) Root and shoot lengths of 30-days-old WT and *OsPAP21b* RNAi lines under +P, (b) -P and (c) +ATP conditions. Plants were raised hydroponically for 30 days under +P, -P and +ATP conditions (15 days Pi-starved seedlings were recovered with ATP for 15 subsequent days). Each values represents mean of 15 seedlings with standard error. Level of significance was analysed with student's *t*-test. * p value < 0.05; ** p value < 0.01; *** p value < 0.001.

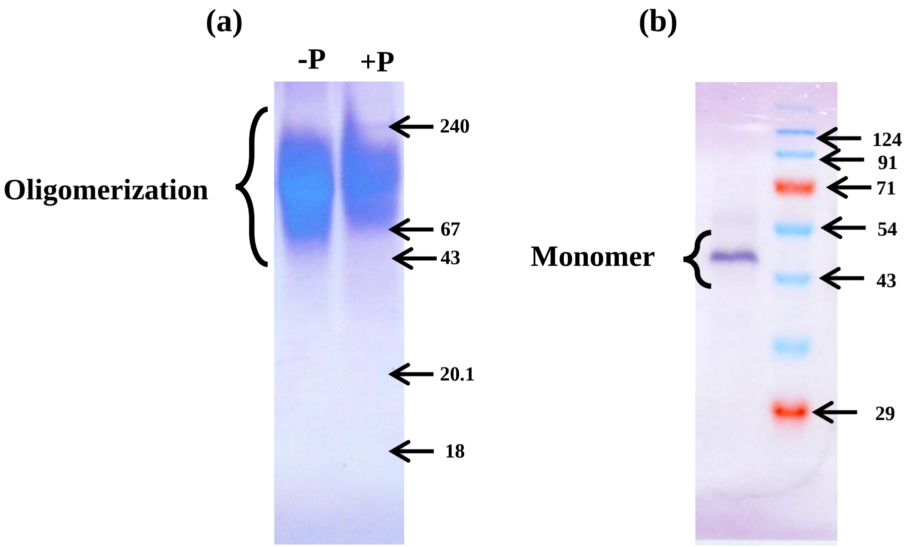

Figure S14. OsPAP21b form high molecular weight complex in plant. (a) Western blot of OsPAP21b showing oligomerization of OsPAP21b with unknown cellular proteins. 24 µg of total protein from OE10 was resolved on 8% non-denaturing PAGE followed by blotting and detection (*see supplementary methods*). Sizes of protein complexes were determined by calibrating pre-stained ladder with native PAGE protein marker (Genei). (b) Western blot of OsPAP21b showing molecular weight of monomeric OsPAP21b (~49 kDa). 24 µg of total protein from OE10 was resolved on 12% denaturing SDS-PAGE followed by western blotting and detection.

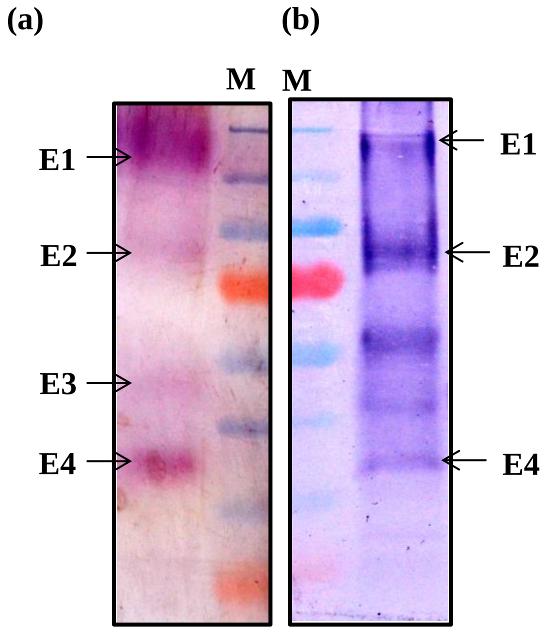

Figure S15. Alignment of APase profile of OE10 with immunoblot of OE10 protein probed with anti-OsPAP21b antibody. (a) 10 µg of total root protein of one-month-old Pi-starved OE10 were separated on 10% non-reducing SDS-PAGE. Different APase isoforms were detected by staining with fast black potassium salt and β-naphthyl acid phosphate (*see methods*). (b) Identical gel was subjected to immunoblotting using anti-OsPAP21b antibody. Different APase isoforms are indicated by arrows.

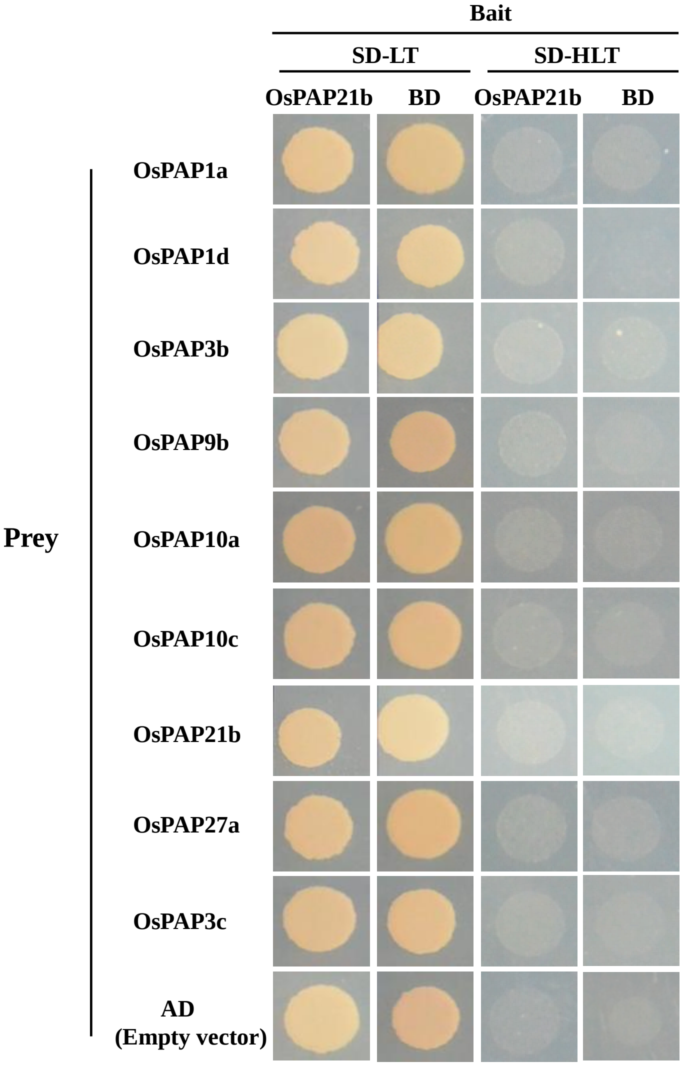

Figure S16. Yeast two-hybrid assays of OsPAP21b with different PSR PAPs. OsPAP21b was cloned in to bait vector (BD), pGBKT7. All PAPs were cloned in prey vector (AD), pGADT7. Both prey and bait were co-transformed in yeast strain Y2H Gold. Transformed clones were selected on SD-LT minimal media and assayed for positive interactions on SD-HLT selection media lacking histidine, leucine and tryptophan.

Supporting methods

Plant phenotyping

Root and shoot lengths were measured manually with ruler. Dry biomass of plants was quantitated after oven drying at 80 ºC (till constant weight). For quantitation of lateral root length and numbers, roots of fifteen-days-old OE lines and wild type (WT) raised hydroponically under +P and –P conditions were photographed with Nikon DSLR 5100. All images were analysed with ImageJ 1.46r (http://imagej.nih.gov/ij). Statistical analysis was carried out using Student's *t*-test.

Quantitative Real-Time PCR

Total RNA was isolated using TRIzol reagent (Ambion) following manufacturer's protocol. Extracted RNA was treated with DNase (1 U/µg RNA). cDNA was synthesized using high-capacity cDNA reverse transcription kit (Applied Biosystems) as per manufacturer's instructions. qRT-PCR was carried out on Applied Bio systems 7500 Fast Real-Time PCR System using SYBR® Green (Applied Biosystems). *Ubiquitin5* (Os01g0328400) was used as endogenous control. Relative expression levels were calculated using $2^{-\Delta(\Delta Ct)}$ method.

Electrophoretic mobility shift assay (EMSA)

Coding region of OsPHR2 was cloned into *Nde*I and *BamH*1 sites of pET28a vector using gene-specific primers (Table S1). Recombinant construct was transformed into *E. coli*, and 6XHis-OsPHR2 protein was induced and purified. For gel-shift assay, 423 bp promoter region of *OsPAP21b* (containing one P1BS element; -421 to -414) was amplified from rice genomic DNA with primers listed in Table S1. Amplified promoter was radiolabelled with [α^{32}P]CTP using Megaprime DNA labeling system (Amersham Biosciences) according to manufacturer's instructions. Assay was performed with 10 ng radiolabelled OsPAP21b probe with or without 1.5

μg purified OsPHR2 protein in a 30 μl reaction (1 μg poly (dI-dC), 30 mM KCl, 15 mM HEPES (pH 8.0), 0.02 mM DTT, 1 mM MgCl2, 0.2 mM EDTA and 0.6% Glycerol). For competition assay, 400-fold excess unlabeled promoter probe was used as competitor in the reaction mixture. All reactions were incubated at 20 °C for 30 min followed by electrophoresis on 4% polyacrylamide gel. Gel was analysed by phosphor-imaging using Typhoon 9210 phosphor imager (GE Healthcare).

Total protein extraction and quantitation of APase activity in roots and shoots

Total protein was extracted from 250 mg root and shoot in chilled extraction buffer as described (Wang *et al*., 2011). For quantitation of total APase activity, 1 µg of total protein was incubated with 10 mM pNPP in reaction mixture containing 5 mM $MgCl_2$ and 50 mM sodium acetate, pH 5.0. Reactions were incubated at 37ºC for 15 min, after which amount of released Pi was measured spectrophotometrically by yellow vanadomolybdate method at 410 nm.

Detection of In-Gel APase profile and mass spectrometry of target protein

In-gel APase profile was analysed as described (Wang *et al*., 2011) with slight modifications. Briefly, 10 µg of total root and shoot proteins were resolved on 10% non-reducing SDS-PAGE gels. Gels were subsequently washed six times with ice-cold distilled water for a total of 1 h (10 min per wash). Thereafter, proteins were renatured by incubating gels in renaturation buffer containing 50 mM sodium acetate, pH 5.0 and 10 mM $MgCl_2$ for 30 min. To visualize different isoforms encoded by APases, gels were stained with renaturation buffer containing 0.5 mg/ml fast black potassium salt and 0.3 mg/ml β-naphthyl acid phosphate at 37°C overnight. For confirming that the overexpressed isoform visible in OE lines is encoded by OsPAP21b, activity-stained non-reducing SDS PAGE gels were aligned with the coomassie-stained non-reducing gels. Coomassie-stained protein bands were digested with trypsin (Sigma) as per manufacturer’s

protocol. Protein samples were subjected to identification by LC-MS/MS using QSTAR Elite mass spectrometer (Applied Biosystems) coupled with an ultimate 3000 HPLC system (Dionex) as described (Jaiswal *et al*., 2013).

Quantitation of secretory APase activity

One-month-old WT and OE lines raised under +P and -P conditions were used for quantitation of secretory APase activity. Individual plants were transferred to culture tubes containing fresh 50 ml +P or -P growth medium. A total of 10 plants per transgenic line were used. Plants were kept at 30°C for 24 h. After this, 800 µl medium was collected from each tube and incubated with 5 mM pNPP at 37°C for 1h. Reaction was stopped by adding 100 µl of 0.4 M NaOH and APase activity was quantitated spectrophotometrically at 410 nm.

For quantitation of secretory APase activity from concentrated protein, ten 15-days-old plants of WT and OE lines were kept in 2 litres of +P medium or –P medium for another 15 days. Whole medium was thereafter filtered with 0.4 µ filter and lyophilized at -50°C. Lyophilized samples were suspended in 2 ml of protein extraction buffer as described above and desalted through PD MidiTrap G-25 columns (GE Amersham). For determination of APase activity, 5 µg of concentrated secretory protein was incubated with 5 mM of pNPP in a 900 µl reaction containing 50 mM sodium acetate (pH 5.0) and 10 mM $MgCl_2$ at 37°C for 1h. Reaction was stopped by adding 100 µl of 0.4 M NaOH and absorbance was measured at 410 nm. Whole experiment was carried out in three replications.

Activity staining of root surface associated APases

Roots were placed on a flat surface and overlaid with 0.015% BCIP (5-bromo-4-chloro-3-indolyl-phosphate) in 0.5% agar solution. Secretory APase activity was visualized as blue colour precipitate on root surface.

Yeast two-hybrid assays

Protein-protein interactions were performed using Matchmaker Gold Yeast two-hybrid system (Clonetech) as per manufacturer's protocol. Coding sequence of *OsPAP21b* was cloned into bait vector (BD), pGBKT7. All other PAPs were cloned into appropriate restriction sites of prey vector (AD), pGADT7. Primers are listed in Table S1. AD clones were cotransformed with OsPAP21b-BD in yeast strain Y2H Gold (Clonetech) and selected on minimal media lacking histidine, leucine and tryptophan (-HLT).

Generation of anti-OsPAP21b antibody and immunoblot analysis

For raising polyclonal antibody against OsPAP21b, two healthy New Zealand white rabbits were immunized with purified recombinant OsPAP21b protein. Antiserum was produced and purified by Link Biotech, India. Specificity of antibody was determined by immunoblotting of purified recombinant OsPAP21b protein (Fig. S1d) and total plant protein (Fig. S14b).

For immunoblotting assays, proteins were resolved on 12% SDS PAGE, 10% non-reducing SDS-PAGE or 8% non-denaturing PAGE and blotted on PVDF (polyvinylidene difluoride) membrane in transfer buffer (25 mM Tris base, 192 mM glycine, pH 8.3) at 4°C for 1 h. Membranes were blocked by incubation with 5% non-fat dry milk in PBST for overnight at 4°C. This was followed by incubation with rabbit anti-OsPAP21b antibody (1:1000) for 2 h at room temperature. Blots were thereafter incubated with goat anti-rabbit antibody conjugated with alkaline phosphatase (1:10000) for 2 h at room temperature. Following every incubation, blots were washed thrice with PBST, consecutively for 5, 10 and 5 min each time. Finally, blots were developed with BCIP/NBT solution (Merck).

Total P content analysis

For quantitation of total P content, 150 mg of oven dried tissue was subjected to ashing at 550°C for 5 hrs in muffle furnace. Total P was extracted by suspending ash in 2 ml of 2N HCl for 20 min. Whole suspension was centrifuged at 13000 rpm for 5 min. 100 µl of supernatant was used for total P measurement by yellow vanadomolybdate method as described in materials and methods.

Subcellular localization of OsPAP21b

Coding region of OsPAP21b was fused with YFP coding sequence under *CaMV35S* promoter in Gateway compatible binary vector, pSITE3CA. Particle bombardment of YFP-OsPAP21b and organelle markers (ABRC); mt-rk (CD3-991), g-rk (CD3-967), pt-rk (CD3-999), px-rk (CD3-983), er-rk (CD3-959), were performed as described earlier (Giri *et al.*, 2011). Images were analysed in AOBS TCS-SP2 (Leica, Germany) confocal microscope. For plasmolysis, onion peels expressing YFP-OsPAP21b were treated with 5 % NaCl for few minutes.

References

Giri, J., Vij, S., Dansana, P.K. and Tyagi, A.K. (2011) Rice A20/AN1 zinc-finger containing stress-associated proteins (SAP1/11) and a receptor-like cytoplasmic kinase (OsRLCK253) interact via A20 zinc-finger and confer abiotic stress tolerance in transgenic Arabidopsis plants. *New Phytol.* **191,** 721-732.

Jaiswal, D.K., Ray, D., Choudhary, M.K., Subba, P., Kumar, A., Verma, J., *et al.* (2013) Comparative proteomics of dehydration response in the rice nucleus: New insights into the molecular basis of genotype-specific adaptation. *Proteomics* **13**, 3478-3497.

Wang, L., Li, Z., Qian, W., Guo, W., Gao, X., Huang, L. *et al.* (2011) The Arabidopsis purple acid phosphatase AtPAP10 is predominantly associated with the root surface and plays an important role in plant tolerance to phosphate limitation. *Plant Physiol.* **157,** 1283-1299.
